# Imaging-mediated genetic effects link brain microstructure, metabolic profiles, and regional transcription to glioma susceptibility

**DOI:** 10.3389/fimmu.2026.1870121

**Published:** 2026-07-03

**Authors:** Yufan Wu, Xuezhen Wang, Xinkai Wang, Lin Chen, Shiqi Huang, Mingwei Zhang, Jinsheng Hong

**Affiliations:** 1 Department of Radiotherapy, Cancer Center, The First Affiliated Hospital, Fujian Medical University, Fuzhou, Fujian, China; 2 Department of Radiotherapy, National Regional Medical Center, First Affiliated Hospital, Fujian Medical University, Fuzhou, Fujian, China; 3 Key Laboratory of Radiation Biology of Fujian Higher Education Institutions, The First Affiliated Hospital, Fujian Medical University, Fuzhou, Fujian, China

**Keywords:** glioma, imaging-derived phenotypes, mediation analysis, metabolites, multi-omics

## Abstract

**Background:**

This study aimed to systematically characterize genetically links and Mediation mechanism between glioma susceptibility and brain microstructure, cross-compartment metabolic profiles, and region-specific gene expression, followed by functional validation.

**Methods:**

Using GWAS data from 12,488 glioma cases and 18,169 controls, we performed two-sample MR (TSMR) and summary data-based MR (SMR) analyses. We evaluated 587 brain imaging-derived phenotypes (IDPs) from diffusion and structural MRI; levels of 962 brain tissue metabolites, 440 cerebrospinal fluid (CSF) metabolites, and 1,400 plasma metabolites; and eQTL based gene expression across 13 brain regions. A two-step MR design was employed for mediation analysis, and the key gene identified, *HEATR3*, underwent clinical correlation and *in vitro* functional validation.

**Results:**

TSMR identified 6 IDPs significantly associated with all glioma subtypes. Specifically, elevated intracellular volume fraction (ICVF) in the corpus callosum and cingulum increased risk, while increased mean diffusivity (MD) in the posterior limb of the right internal capsule was protective. Integrated SMR and TSMR revealed that elevated *HEATR3* expression across all 13 brain regions significantly increases glioma risk. Functional experiments confirmed *HEATR3* is upregulated in glioma, correlates with poor patient prognosis, and promotes malignant progression *in vitro*. Mediation analysis showed that *HEATR3*’s effect on glioma risk is partially mediated by specific white matter IDPs. Metabolic analysis revealed that higher levels of orotate—a product of *de novo* pyrimidine synthesis—in plasma, CSF, and brain tissue significantly increase GBM risk. Mediation analysis suggested that the effect of plasma orotate on glioma risk is partially mediated by the ICVF in the right cingulum hippocampus. Enrichment analyses indicated that significant regional genes are heavily involved in DNA metabolism and cell cycle pathways.

**Conclusion:**

This study provides robust multi-layered evidence for the causal roles of white matter microstructural abnormalities, metabolic dysregulation, and regional gene expression in glioma development. These findings offer novel genetic insights and potential therapeutic targets for precision medicine in glioma.

## 1 Introduction

Glioma is the most common primary malignant brain tumor in adults, accounting for approximately 80% of all cases (1). Despite continuous innovations in clinical treatments, the overall prognosis for glioma remains poor; even with standard therapy, the median survival for glioblastoma (GBM), the most common subtype, is only 12 to 15 months (2). The etiology of glioma remains unclear. Although exposure to ionizing radiation (3, 4) and rare genetic syndromes such as neurofibromatosis (5) are known risk factors, they account for only a small minority of cases, leaving the causes of the vast majority of gliomas yet to be explored. Existing studies have largely focused on lifestyle and dietary factors proven to affect the risk of other cancers (6, 7), while long neglecting the intrinsic characteristics of the host organ itself—the brain.

The emerging field of cancer neuroscience is dedicated to uncovering the complex interactions between brain tumors and the structural and functional networks of the brain (8). Structural and diffusion MRI in neuroimaging can quantitatively reflect gray and white matter volumes, cortical area and thickness, as well as the microstructural features of white matter tracts; these phenotypes are influenced not only by development and aging but are also closely related to genetics (9, 10). These image-derived phenotypes (IDPs) provide a non-invasive, quantitative means to reveal the structural and microstructural characteristics of the brain, offering a valuable data source for exploring brain-disease associations (11). In recent years, studies have begun to focus on the association between brain IDPs and various diseases, suggesting that the structural or functional state of the brain may influence susceptibility to neurological and systemic diseases (12–14). However, systemic evidence regarding the causal relationship between these IDPs and glioma risk is currently lacking.

On the other hand, metabolic reprogramming is a core hallmark of cancer. Glioma cells exhibit unique metabolic profiles to meet the energy and biosynthetic demands of rapid proliferation, which are genetically regulated (15) and can be linked to brain function and structure (16). For instance, the abnormal accumulation of the metabolite D-2HG driven by IDH mutations promotes tumorigenesis by affecting epigenetic regulation and cell differentiation, which can be quantitatively measured via 3D magnetic resonance spectroscopic imaging (MRSI) (17). In normal cerebral white matter, the ratio of N-acetylaspartate is significantly positively correlated with diffusion tensor imaging parameters such as fractional anisotropy. However, in the glioma parenchyma and peritumoral edema regions (18), this normal metabolism-structure correlation is disrupted, indicating a loss of normal association between neural function and microstructure in the tumor area.

Notably, the blood-brain barrier (BBB) partially isolates the central nervous system from the peripheral metabolic environment (19); however, in high-grade gliomas, BBB disruption may allow a massive influx of plasma metabolites into the tumor interstitium, thereby forming a distinct extracellular metabolome (20). Nevertheless, most studies have focused either on metabolic changes within the tumor tissue itself or have been limited to a single biofluid, such as CSF or plasma (21, 22). A combined analysis of brain tissue, CSF, and plasma metabolites is crucial for comprehensively understanding metabolic dysregulation in glioma. Furthermore, different brain regions possess unique gene expression patterns that form the molecular basis of their functional differentiation (23), profoundly impacting regional brain functions (24–26). Given the complex crosstalk among gene expression, metabolic processes, and brain structure, studying these aspects in isolation may overlook key interactions and miss critical mechanistic insights. Therefore, a multi-omics integrative approach is essential to unveil the underlying pathophysiological mechanisms of glioma.

Mendelian randomization (MR) is a robust epidemiological tool that utilizes genetic variants as instrumental variables to infer causal relationships between exposures and outcomes (27). Because genetic variants are not influenced by acquired environmental factors, MR studies can effectively circumvent the potential confounding biases inherent in traditional observational research. This study employs TSMR to systematically evaluate the causal associations of IDPs and multi-tissue metabolites (including brain tissue, CSF, and plasma) with glioma and its subtypes (GBM and non-GBM), followed by a mediation analysis to explore the potential mediating pathways of IDPs and metabolites in glioma pathogenesis. Building upon this, we further integrate the SMR approach to analyze the causal effects of regional brain gene expression on glioma risk, accompanied by clinical correlation analyses and experimental validation of the identified key genes. This study aims to provide novel multi-layered evidence for the etiology of glioma from a genetic perspective and to lay a theoretical foundation for developing imaging- and metabolomics-based early risk prediction strategies and novel therapeutic targets in the future.

## 2 Materials and methods

### 2.1 Study design

**Figure 1:**
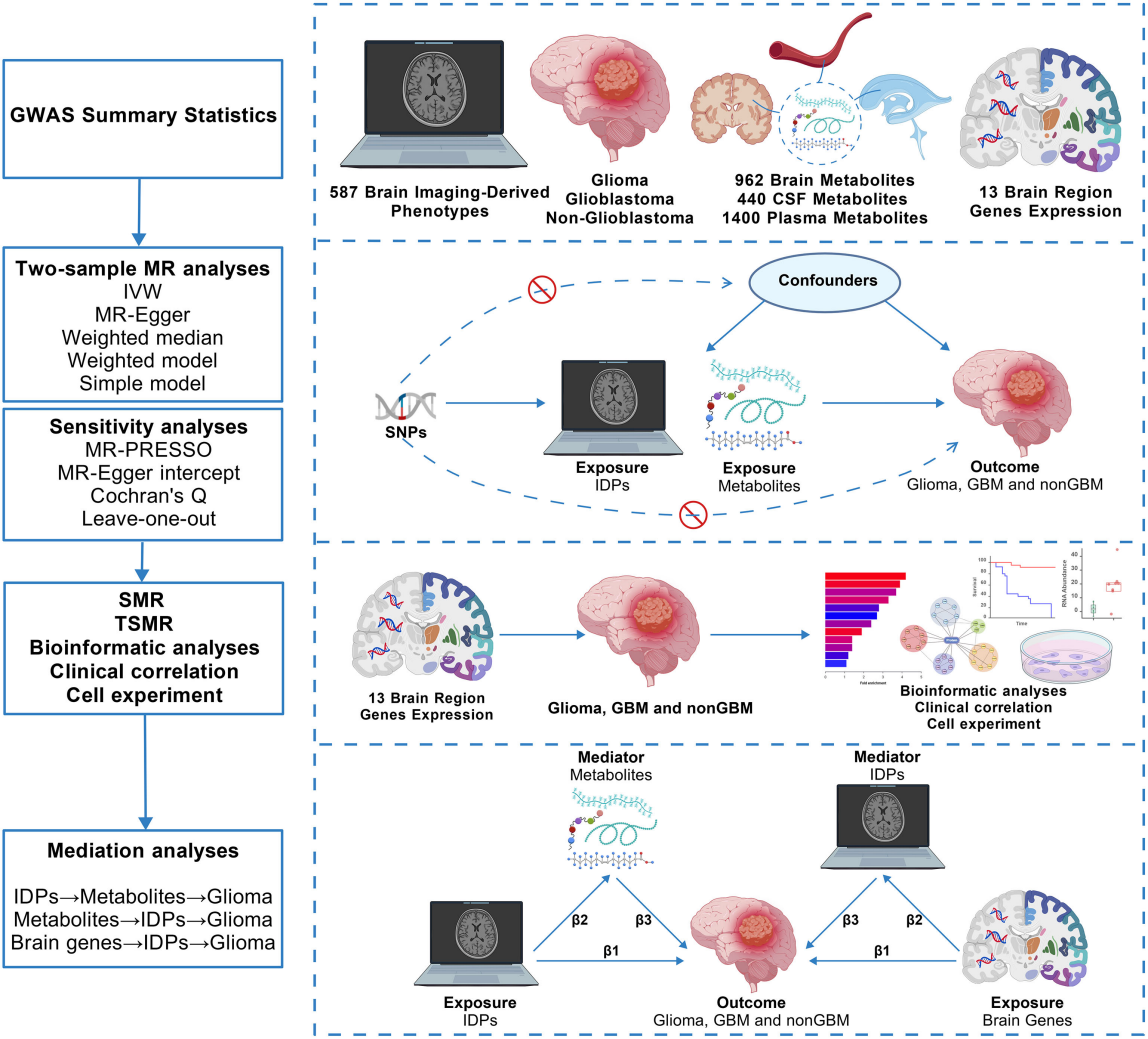
Flowchart of the overall study design.

This study employed a comprehensive multi-omics Mendelian randomization analysis framework to systematically explore the causal pathways and mediating mechanisms underlying the associations of IDPs, metabolites in brain tissue, CSF, and plasma, as well as regional brain gene expression, with glioma pathogenesis. **Figure 1** outlines the overall study design, which primarily consists of the following three core components:

First, two-sample MR (TSMR) was utilized to evaluate the causal effects of IDPs, as well as metabolites in brain tissue, CSF, and plasma, on the risk of overall glioma, GBM, and non-GBM subtypes. TSMR and summary data-based MR (SMR) methods were used to assess the impact of regional brain gene expression on glioma, aiming to identify key genes with potential pathogenicity.

Second, based on these results, a two-step MR mediation analysis was conducted to uncover the potential mediating pathways of IDPs, metabolites, and regional brain genes in glioma development.

Finally, genes demonstrating significant causal associations with glioma were subjected to Gene Ontology (GO) and Kyoto Encyclopedia of Genes and Genomes (KEGG) pathway enrichment analyses, as well as protein-protein interaction (PPI) network construction. For key genes showing strong causal effects across multiple brain regions, survival and prognostic analyses were performed using clinical databases, and *in vitro* cellular functional experiments were carried out to verify their impact on the malignant phenotypes of glioma cells.

### 2.2 Data sources

#### 2.2.1 Glioma GWAS data

GWAS data for glioma were obtained from the largest glioma meta-GWAS analysis to date (28), which integrated eight independent GWAS datasets. The summary data included a total of 6,183 GBM cases, 5,820 non-GBM cases, and 18,169 control individuals of European descent (**Supplementary Table 1**).

#### 2.2.2 Brain imaging-derived phenotype GWAS data

Summary data for brain IDPs were acquired from the IEU OpenGWAS project, which derived from a genome-wide association analysis of 33,224 participants in the UK Biobank (29). The 587 IDPs used in this study originated from two magnetic resonance imaging modalities: structural MRI (sMRI), used to describe brain anatomy; and diffusion MRI (dMRI), used to assess the microstructural and connectivity properties of white matter tracts. All IDPs were categorized into 13 brain region classes and 9 measurement metric types. The 13 brain regions encompassed 8 anatomical areas and 5 structural connectivity tracts. Regarding measurement metrics, 3 types of metrics reflecting brain anatomical features—area, thickness, and volume—were extracted based on sMRI. Six types of diffusion metrics reflecting white matter microstructure were extracted based on dMRI, including fractional anisotropy (FA), mean diffusivity (MD), mode of anisotropy (MO), intracellular volume fraction (ICVF), isotropic volume fraction (ISOVF), and orientation dispersion index (OD) (**Supplementary Table 2**).

#### 2.2.3 Metabolite GWAS data

GWAS summary data for brain and CSF metabolites were obtained from a metabolome-wide association study conducted by Cruchaga et al. on 2,602 CSF samples and 1,016 brain tissue samples (30), covering genetic information for 440 CSF metabolites and 962 brain tissue metabolites (**Supplementary Table 3**).

Plasma metabolite data were sourced from the Canadian Longitudinal Study on Aging (CLSA) cohort (31), which included GWAS summary results for 1,091 metabolites and 309 metabolite ratios from 8,299 participants (**Supplementary Table 4**).

#### 2.2.4 Brain regional gene expression eQTL data

eQTL Data for gene expression across 13 brain regions were provided by the GTEx Consortium (https://gtexportal.org/home/datasets). The included brain regions were: Amygdala (B1), Anterior cingulate cortex BA24 (B2), Caudate basal ganglia (B3), Cerebellar Hemisphere (B4), Cerebellum (B5), Cortex (B6), Frontal Cortex BA9 (B7), Hippocampus (B8), Hypothalamus (B9), Nucleus accumbens basal ganglia (B10), Putamen basal ganglia (B11), Spinal cord cervical c-1 (B12), and Substantia nigra (B13).

### 2.3 Selection of instrumental variables

To satisfy the fundamental assumptions of MR analysis, all instrumental variables (IVs) underwent rigorous screening. First, single nucleotide polymorphisms (SNPs) significantly associated with the exposure factors at the genome-wide significance level (p < 5×10^-8^) were preferentially selected. If fewer than 3 SNPs met this threshold, the criterion was relaxed to p < 5×10^-6^ to ensure a sufficient number of IVs. Subsequently, linkage disequilibrium among SNPs was evaluated using the “clump data” tool, setting a window size of 10,000 kb and an r² threshold of 0.001 to exclude highly linked SNPs and ensure the independence of IVs. All non-biallelic SNPs and palindromic SNPs were also removed. To assess weak instrument bias, the F-statistic was calculated. Additionally, by querying the GWAS Catalog (https://www.ebi.ac.uk/gwas), SNPs associated with known confounding factors were identified and excluded to strengthen the specificity of the pathways linking IVs to the outcome.

### 2.4 TSMR analysis

TSMR analysis relies on three key assumptions (1): IVs are robustly associated with the exposure (2); IVs are independent of confounding factors; and (3) IVs influence the outcome exclusively through the exposure. These assumptions form the foundation for the validity of MR causal inference (27).

To comprehensively evaluate the causal relationships between IDPs, multi-tissue metabolites, and glioma, we employed various MR methods, including inverse-variance weighted (IVW), MR-Egger regression, simple mode, weighted mode, and weighted median. The IVW method served as the primary analytical approach, given its robust performance when IVs are valid and pleiotropy is balanced (32).

Results were reported as odds ratios (ORs) with their 95% confidence intervals (CIs) and p-values. The criteria for determining statistical significance were as follows: if the IVW p-value remained < 0.05 after false discovery rate (FDR) correction, and there was no evidence of horizontal pleiotropy or heterogeneity, a statistically robust causal relationship was deemed to exist. If the IVW p-value was < 0.05 and the results were consistent across all sensitivity analyses but did not survive FDR correction, it was considered a potential causal effect.

### 2.5 Sensitivity analysis

To systematically assess the robustness and consistency of the MR results, we employed multiple sensitivity analysis methods. Cochran’s Q test was used to evaluate heterogeneity among IVs, with p > 0.05 indicating no significant heterogeneity. The intercept term of MR-Egger regression was tested to evaluate horizontal pleiotropy, with p > 0.05 indicating no significant evidence of pleiotropy. The MR-PRESSO method was utilized to identify and remove outlier SNPs that might introduce bias, and a leave-one-out analysis was performed to determine whether the results were disproportionately driven by any single IV.

### 2.6 SMR analysis of regional gene expression in the brain

We employed the SMR method to assess the potential causal effects of genes expressed in 13 distinct brain regions on glioma and its subtypes. eQTL data from different brain regions, obtained from the GTEx Consortium, were used as proxy indicators for gene expression. Screening criteria included a minor allele frequency (MAF) > 1% and a significance of p < 5×10^-8^, restricting the analysis to *cis*-eQTLs. The significance threshold was set at P < 0.05, and the results were required to pass the HEIDI test to support the presence of a shared causal variant rather than an association driven by linkage disequilibrium (33).

The analysis identified several regional brain genes whose expression likely exerts a causal effect on glioma risk, with several target genes commonly implicated in both GBM and non-GBM subtypes. For these genes, we conducted further bioinformatics analyses, including GO functional enrichment, KEGG pathway enrichment, and PPI network construction (https://string-db.org/). To validate the SMR results and enhance methodological robustness, we also applied the TSMR method, using the same regional brain eQTL data as exposure IVs, to analyze the impact of regional gene expression on glioma risk. The results from both methods corroborated each other.

### 2.7 Mediation analysis

We applied a two-stage, two-sample Mendelian randomization framework to explore whether IDPs mediated the effects between regional brain genes, metabolites, and glioma. First, based on the preliminary MR results, we selected IDPs, metabolites, and regional brain genes that demonstrated significant causal associations with overall glioma, GBM, or non-GBM, and lacked evidence of heterogeneity or pleiotropy. Second, bi-directional two-sample MR analyses were conducted to evaluate the potential impact of metabolites or regional genes on the IDPs. Finally, candidate mediating pathways were screened based on effect directions ensuring logical consistency: letting β_1_ represent the effect of the exposure on the outcome, β_2_ the effect of the exposure on the mediator, and β_3_ the effect of the mediator on the outcome. If β_1_ was positive, β_2_ and β_3_ were required to have the same sign; if β_1_ was negative, β_2_ and β_3_ were required to have opposite signs. The mediation effect was calculated using the product of coefficients method (β_2_ × β_3_), and the mediation proportion was estimated as (β_2_ × β_3_)/β_1_ (34).

### 2.8 Enrichment analysis of metabolites

Using the KEGG database, enrichment analyses were performed on the identified CSF and brain metabolites to elucidate metabolic pathways potentially related to the biological processes of glioma.

### 2.9 Clinical correlation analysis

For the identified genes of interest, we acquired transcriptome data of glioma and normal brain tissues from public databases, including glioma samples from The Cancer Genome Atlas (TCGA), the Chinese Glioma Genome Atlas (CGGA), and the Rembrandt database, as well as normal brain tissue data from GTEx. Standardized pipelines were used to process the RNA-seq data, normalized as FPKM, to compare gene expression levels between glioma and normal brain tissues. Expression differences across various WHO pathological grades were also compared. Based on gene expression levels, patients were stratified into high- and low-expression groups. Overall survival was analyzed using the Kaplan-Meier method, and inter-group differences were compared via the Log-rank test. The infiltration level of different immune cell types in every sample was estimated using CIBERSORTx (https://cibersortx.stanford.edu/).

### 2.10 Cell experiments

For detailed methods of the cell experiments, please refer to the **Supplementary Material**.

## 3 Results

### 3.1 Causal effects of brain imaging phenotypes on glioma

**Figure 2:**
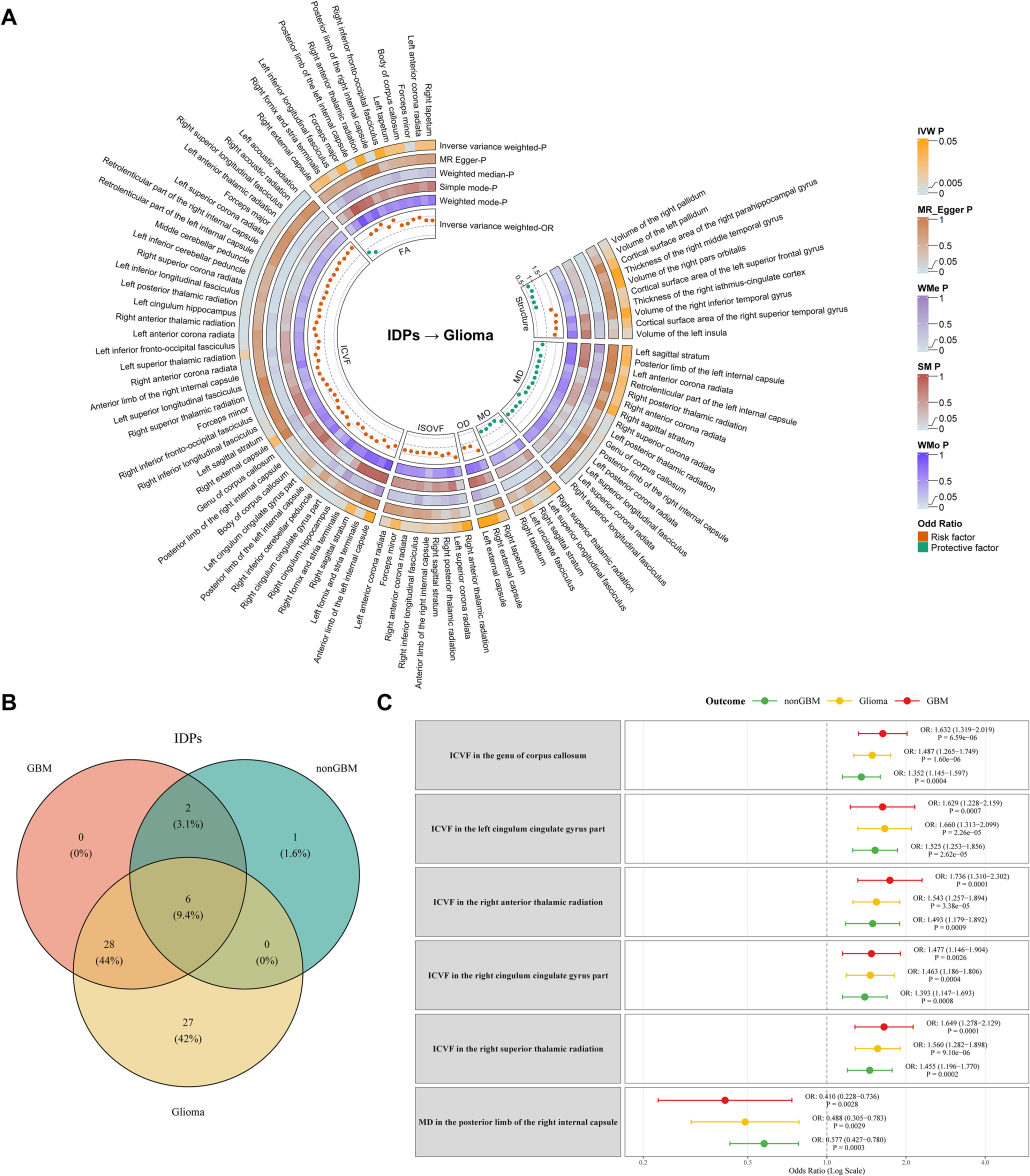
Mendelian randomization analysis of causal effects of IDPs on glioma. **(A)** Circular heatmap showing the causal effects of IDPs on overall glioma. **(B)** Venn diagram illustrating IDPs with significant causal effects on overall glioma, GBM, and nonGBM. **(C)** Forest plot depicting the causal effects of IDPs significant for overall glioma, GBM, and nonGBM.

This study systematically evaluated the causal relationships between IDPs and glioma, including its subtypes. At the significance level of IVW p < 0.05, 94 IDPs were causally associated with overall glioma, 102 with GBM, and 64 with non-GBM. Following FDR correction, 36 IDPs remained causally associated with overall glioma, 61 with GBM, and 9 with non-GBM. Overall, these causal associations were predominantly concentrated in diffusion metrics, including ICVF, ISOVF, FA, and MD. A Venn diagram identified 6 IDPs demonstrating consistent associations across overall glioma, GBM, and non-GBM. Specifically, ICVF in the genu of the corpus callosum, ICVF in the left cingulum cingulate gyrus part, ICVF in the right anterior thalamic radiation, ICVF in the right cingulum cingulate gyrus part, and ICVF in the right superior thalamic radiation significantly increased the risk of overall glioma, GBM, and non-GBM; conversely, MD in the posterior limb of the right internal capsule exhibited a protective effect against all three categories (**Figure 2**, **Supplementary Figure 1**) (**Supplementary Table 5**).

Among the structural metrics, cortical surface area of the left superior frontal gyrus increased the risk of non-GBM (OR = 1.885, 95% CI: 1.340-2.653, p = 0.0002), whereas the volume of the left pallidum acted as a protective factor for GBM (OR = 0.663, 95% CI: 0.502-0.876, p = 0.0037).

### 3.2 Causal relationships between regional gene expression and glioma

**Figure 3:**
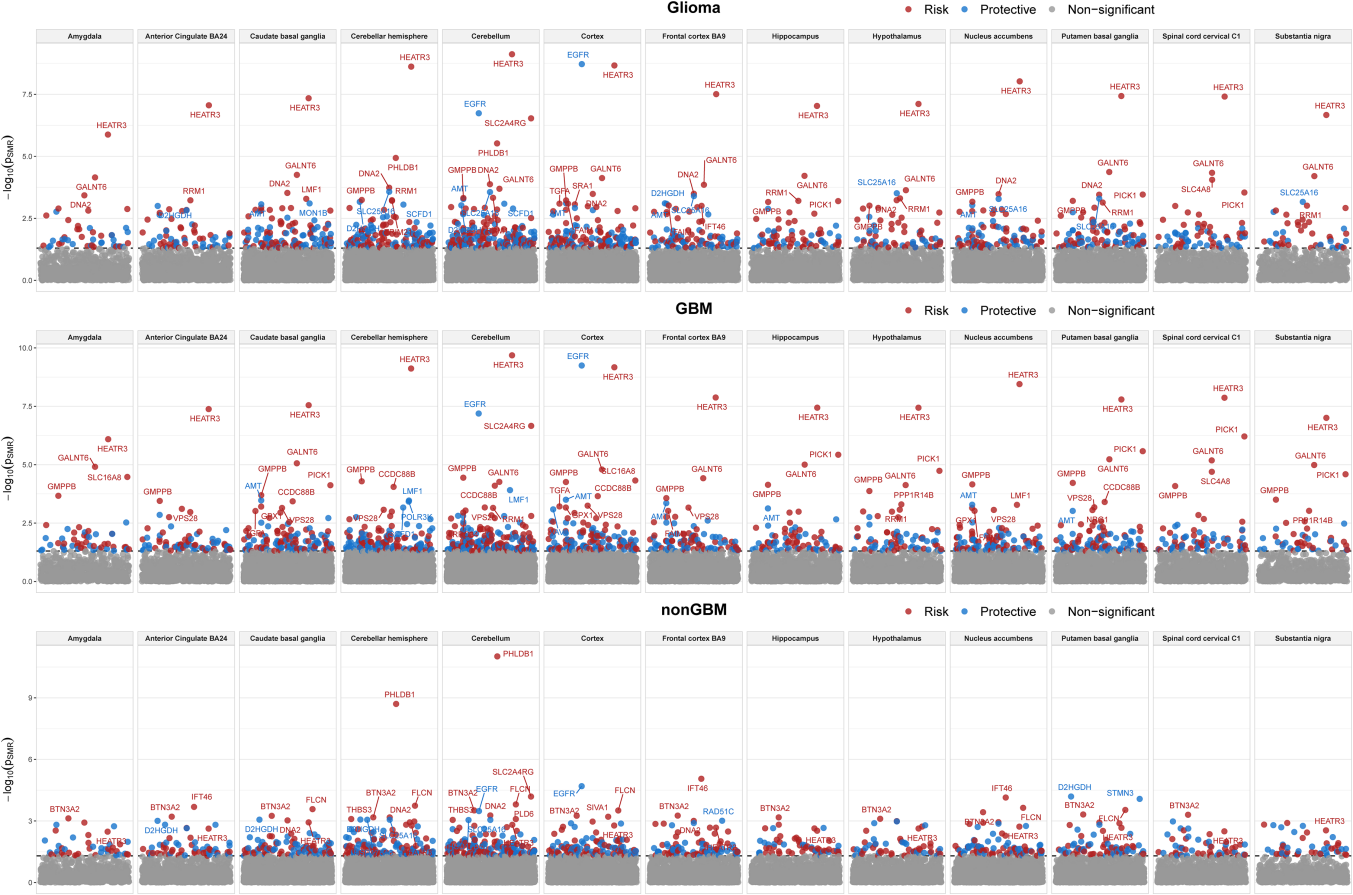
Manhattan plot of SMR results for gene expression in 13 brain regions across glioma, GBM and nonGBM.

Previous TSMR analyses identified IDPs causally related to glioma, some localized to distinct brain regions and others broadly distributed across multiple regions. Based on this observation, we hypothesized that individual brain regions might exert specific or pan-regional effects on glioma risk. To investigate this, we performed a comprehensive SMR analysis using *cis*-eQTL data from 13 brain regions to evaluate the causal associations between regional gene expression and overall glioma, GBM, and non-GBM (**Figure 3**).

**Figure 4:**
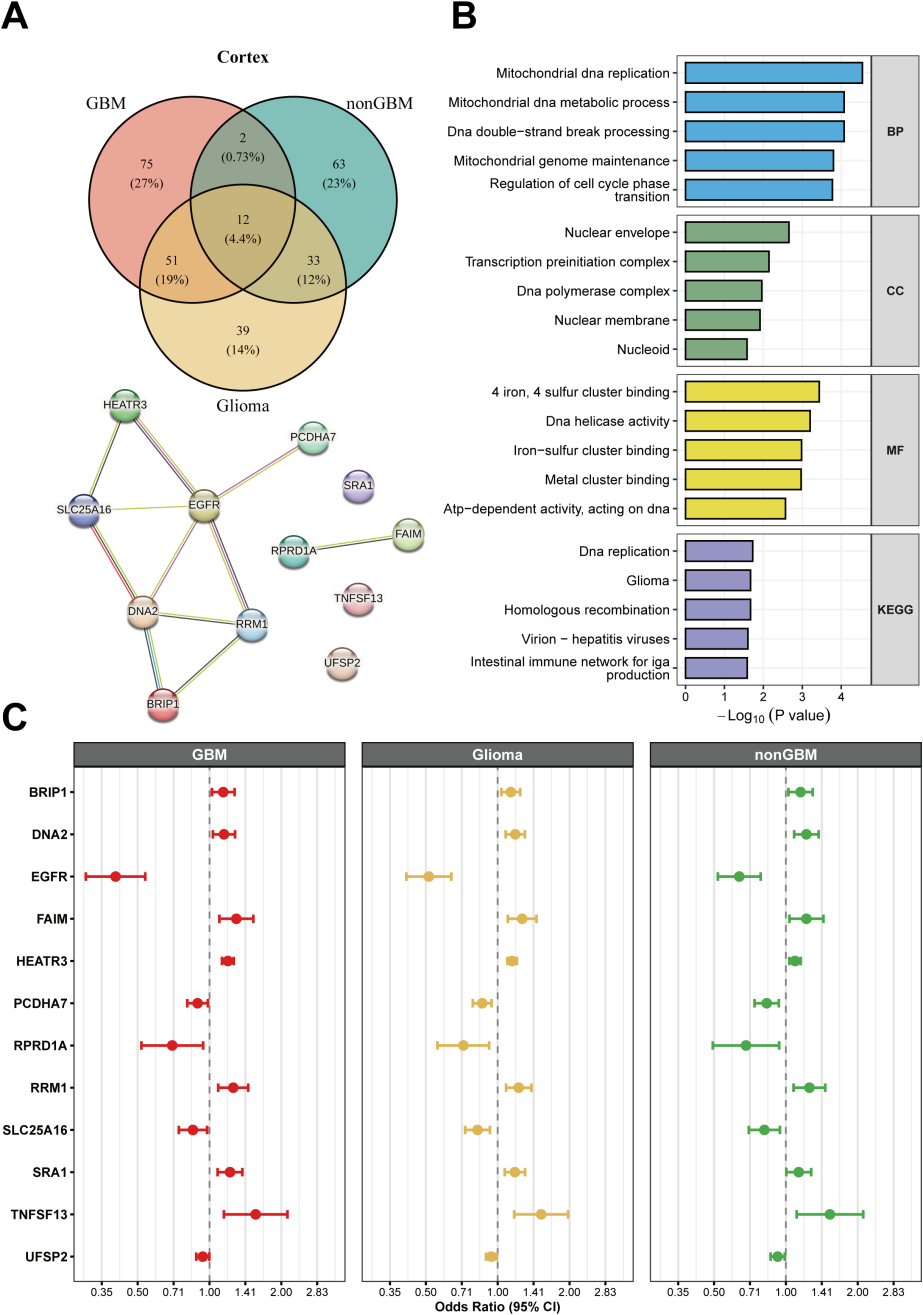
The causal effects of gene expression in Cortex on glioma. **(A)**. Venn diagram and PPI network illustrating gene expression in cortex with significant causal effects on glioma, GBM, and nonGBM. **(B)**. GO and KEGG pathways enriched by genes expressed exhibiting significant causal effects on glioma, GBM, and nonGBM. **(C)**. Forest plot depicting the causal associations of genes expression significant for overall glioma, GBM, and nonGBM.

This analysis identified 135 genes expressed in the cerebral cortex demonstrating causal effects on overall glioma, 140 on GBM, and 110 on non-GBM, with 12 genes shared across all three groups. Among these, 7 genes (*BRIP1, DNA2, FAIM, HEATR3, RRM1, SRA1, TNFSF13*) were associated with an increased risk of glioma and its subtypes, while 5 genes (*EGFR, PCDHA7, RPRD1A, SLC25A16, UFSP2*) exhibited a protective effect, reducing the risk of glioma and its subtypes (**Figure 4A, C**). The PPI network suggested potential functional interactions among these genes (**Figure 4B**). Further GO and KEGG functional enrichment analyses revealed that the genes identified in the cortical region were significantly enriched in DNA metabolism and cell cycle-related pathways, such as regulation of cell cycle phase transition, 5’-3’ DNA helicase activity, positive regulation of DNA replication, nuclear membrane, and homologous recombination (**Figure 4D**).

Forest plots of SMR results for the remaining 12 brain regions, along with corresponding PPI networks and functional enrichment results, are provided in **Supplementary Material**.

Notably, some genes exhibited significant causal effects on overall glioma, GBM, and non-GBM across multiple brain regions. Specifically, the *HEATR3* gene emerged as a risk factor in all 13 brain regions analyzed. These findings suggest that such genes may play pivotal roles in glioma susceptibility and may participate in disease pathogenesis by influencing multiple functional networks globally across the brain. Complete SMR analysis results are detailed in **Supplementary Table 6**.

To validate the robustness of the aforementioned SMR results, we re-evaluated the 13 brain regions using the TSMR method. The results indicated that elevated *HEATR3* expression was significantly causally associated with an increased risk of glioma across all 13 brain regions, which was highly consistent with the SMR findings (**Supplementary Figure 2**). Complete TSMR analysis results are detailed in **Supplementary Table 7.**

### 3.3 Experimental validation of *HEATR3* as a key oncogene in glioma

Given that SMR analysis suggested a widespread causal association between *HEATR3* and glioma risk, yet its role in glioma remained unstudied, we systematically evaluated the clinical significance and biological function of this gene in glioma.

**Figure 5:**
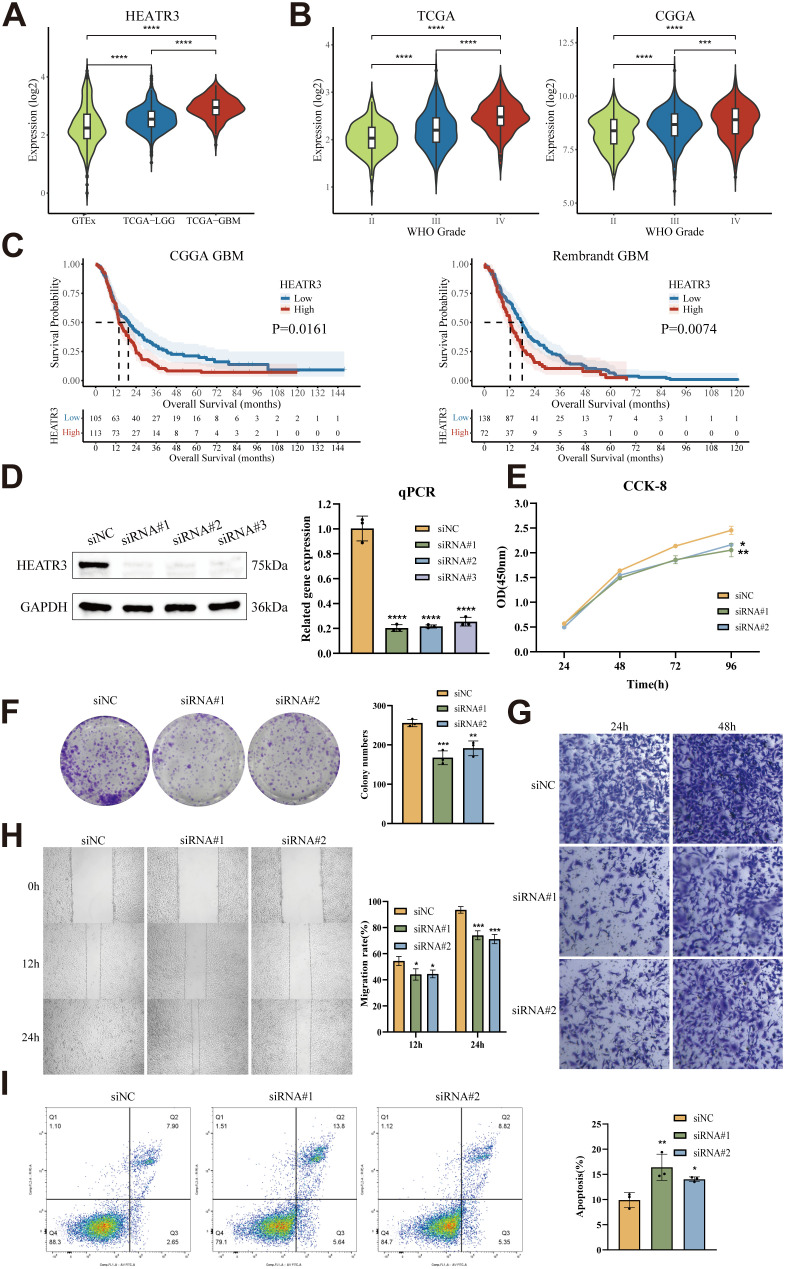
Expression and functional validation of HEATR3 in glioma. **(A)**. Expression of *HEATR3* in normal brain tissues, LGG, and GBM. **(B)**. *HEATR3* expression across WHO grade II, III, and IV gliomas in the TCGA and CGGA cohorts. **(C)**. Kapla-Meier survival curves of GBM patients stratified by *HEATR3* expression in the CGGA and Rembrandt datasets. **(D)**. Western blot and qPCR validation of *HEATR3* knockdown efficiency in U251 glioma cells using siRNA. **(E)**. CCK-8 assay assessing cell viability after *HEATR3* knockdown. **(F)**. Colony formation assay showing proliferative capacity after *HEATR3* knockdown. **(G)**. Transwell assay evaluating cell migration after *HEATR3* knockdown. **(H)**. Wound healing assay demonstrating cell migration ability after *HEATR3* knockdown. **(I)**. Flow cytometry analysis of apoptosis using Annexin V/PI staining following *HEATR3* knockdown. Data are presented as mean ± SD from at least three independent experiments. *p < 0.05, **p < 0.01, ***p < 0.001, ****p < 0.0001 vs. control group.

To define the expression profile of *HEATR3* in glioma, we integrated and analyzed glioma sample data from TCGA, CGGA, and Rembrandt, alongside normal brain tissue data from GTEx. The results showed that *HEATR3* expression levels in glioma tissues were significantly higher than those in normal brain tissues (**Figure 5A**). Further stratified analysis by pathological grade revealed that *HEATR3* expression progressively upregulated with increasing tumor malignancy, indicating a close relationship with tumor progression (**Figure 5B**). In survival analyses, both the CGGA and Rembrandt cohorts demonstrated that patients with high *HEATR3* expression had a significantly shorter OS (CGGA: P = 0.0161; Rembrandt: P = 0.0074) (**Figure 5C**), indicating that high *HEATR3* expression is a risk factor for poor prognosis in glioma patients. Furthermore, *HEATR3* expression exhibited a significant positive correlation with the infiltration of immune cells, including M2 macrophages, neutrophils, and plasma cells, as well as the expression of immune checkpoints such as CD276, IDO1, and LAG3 (**Supplementary Figure 3**).

To deeply validate the biological function of *HEATR3*, we successfully knocked down its expression in the human glioma cell line U251 using siRNA (**Figure 5D**). Functional assays demonstrated that knocking down *HEATR3* significantly inhibited glioma cell proliferation (**Figure 5E**), attenuated cell migration (**Figure 5F**) and invasion capabilities (**Figure 5G**), and reduced colony formation ability (**Figure 5H**). Moreover, flow cytometry revealed that *HEATR3* knockdown promoted cell apoptosis (**Figure 5I**).

### 3.4 Causal effects of metabolites on glioma

Given that regional brain genes were significantly enriched in DNA metabolism-related pathways, indicating that disrupted DNA metabolic homeostasis might play a vital role in glioma pathogenesis, we systematically evaluated the causal associations between circulating and central nervous system metabolites and glioma risk using brain tissue, CSF, and plasma metabolite GWAS data.

**Figure 6:**
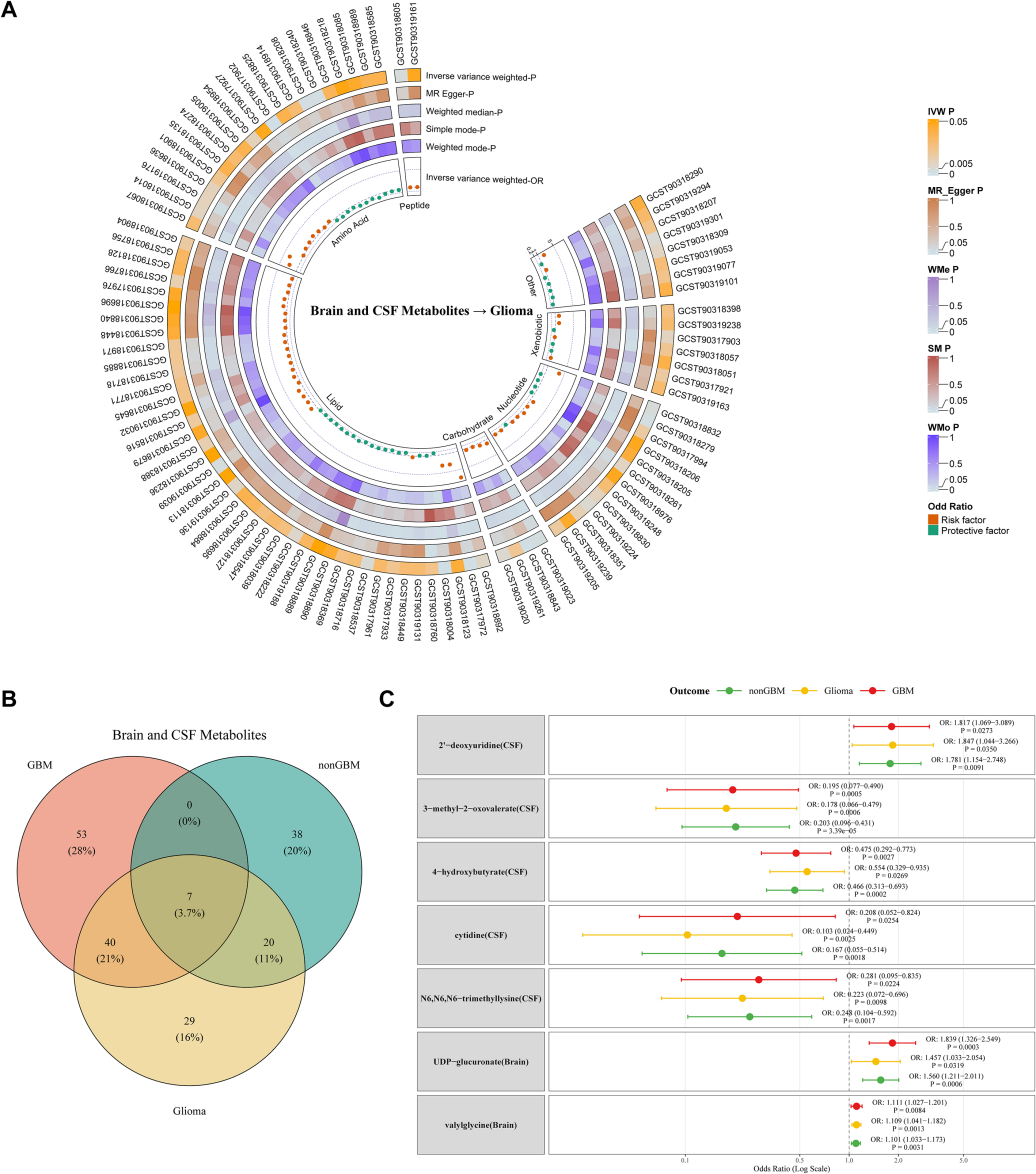
Mendelian randomization analysis of the causal effects of brain and CSF metabolites on glioma. **(A)**. Circular heatmap showing the causal effects of brain and CSF metabolites on overall glioma. **(B)**. Venn diagram illustrating brain and CSF metabolites with significant causal effects on overall glioma, GBM, and nonGBM. **(C)**. Forest plot depicting the causal effects of brain and CSF metabolites significant for overall glioma, GBM, and nonGBM.

TSMR analysis assessed the potential causal relationships between brain tissue and CSF metabolites and glioma. Using the IVW method with a significance threshold of p < 0.05, 96 metabolites were causally associated with overall glioma, 100 with GBM, and 65 with non-GBM. Seven metabolites exhibited consistent effect directions across all three groups. Among them, 3-methyl-2-oxovalerate, 4-hydroxybutyrate, N6,N6,N6-trimethyllysine, and cytidine in the CSF demonstrated a protective effect; whereas 2’-deoxyuridine in the CSF, along with valylglycine and UDP-glucuronate in brain tissue, increased the risk across all three glioma categories. After FDR correction, 3-methyl-2-oxovalerate in the CSF remained a significant protective factor against overall glioma (OR = 0.203, 95% CI: 0.096–0.431, p < 0.0001) (**Figure 6**, **Supplementary Figure 4**) (**Supplementary Table 8**).

KEGG pathway enrichment analysis of these metabolites revealed brain metabolic signatures associated with glioma and its subtypes. Notably, the ABC transporters pathway was consistently and significantly enriched across overall glioma, GBM, and non-GBM, suggesting its universal importance in glioma metabolic regulation. Among GBM-specific enriched pathways, Necroptosis and Efferocytosis were the most prominent. Additionally, GBM showed significant enrichment in multiple metabolism-related pathways, including Choline metabolism in cancer, Sphingolipid signaling pathway, and Pyrimidine metabolism. In contrast, non-GBM displayed a distinct metabolic profile: Central carbon metabolism in cancer and HIF-1 signaling pathway were highly enriched. Concurrently, several amino acid metabolism pathways, such as Valine, leucine and isoleucine biosynthesis, Arginine biosynthesis, and Taurine and hypotaurine metabolism, also showed specific enrichment in non-GBM (**Supplementary Figure 5**).

Similarly, TSMR analysis in plasma identified 88 metabolites and metabolite ratios causally associated with overall glioma, 75 with GBM, and 70 with non-GBM. Eight metabolites were shared among all three categories. The Adenosine 5’-monophosphate (AMP) to urate ratio and X-12830 exhibited protective effects against overall glioma, GBM, and non-GBM. Conversely, 2-hydroxyglutarate, Glucuronide of C12H22O4, Hypotaurine to taurine ratio, Imidazole propionate, Orotate, and Tridecenedioate (C13:1-DC) increased the risk of all three glioma categories (**Supplementary Figure 6**) (**Supplementary Table 9**).

Importantly, we observed that levels of Orotate across plasma (OR = 1.108, 95% CI: 1.039-1.181; p = 0.002), CSF (OR = 1.718, 95% CI: 1.055-2.795; p = 0.029), and brain tissue (OR = 1.382, 95% CI: 1.004-1.902; p = 0.047) universally increased the risk of GBM. (**Supplementary Table 10**).

### 3.5 Mediation analysis

**Figure 7:**
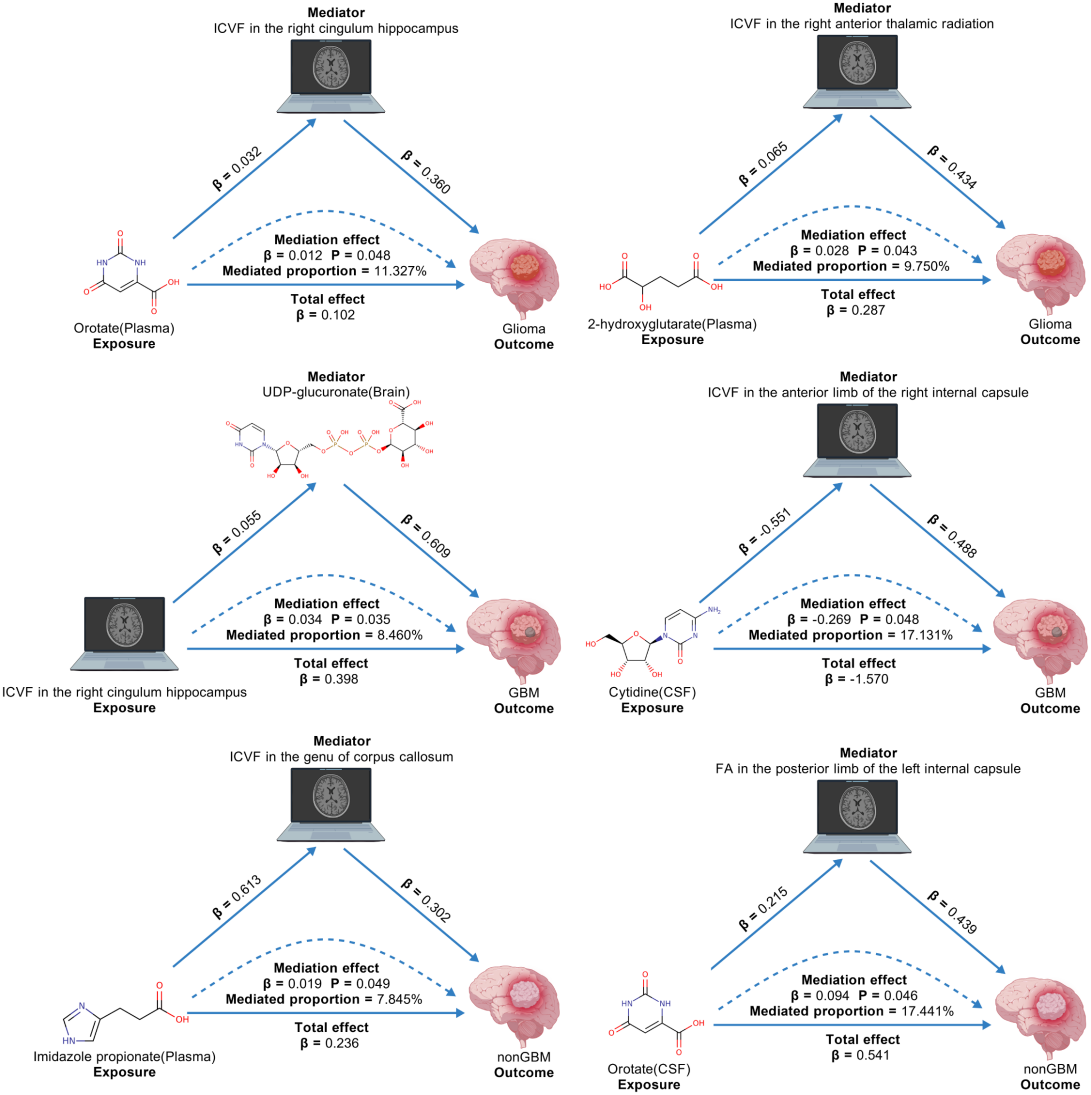
Mediation analysis reveals cross-omics causal pathways linking IDPs, metabolites, and glioma.

Subsequently, we employed a two-step TSMR framework to investigate whether IDPs mediated the associations between metabolites and glioma, and conversely, whether metabolites mediated the causal impact of IDPs on glioma risk. TSMR analyses were conducted on IDPs and metabolites that showed IVW p < 0.05 in previous analyses. By further filtering results based on effect directions and logical consistency of causality, we ultimately identified six mediation pathways with significant mediating effects (p < 0.05) (**Figure 7**).

For overall glioma, the causal effect of plasma orotate on disease risk was partially mediated by ICVF in the right cingulum hippocampus, accounting for a mediation proportion of 11.33%. Additionally, plasma 2-hydroxyglutarate exhibited an indirect effect on glioma risk, which was mediated by the ICVF in the right anterior thalamic radiation, accounting for 9.75% of the total effect.

In GBM, cytidine in the CSF exerted its pathogenic effect by altering the ICVF in the anterior limb of the right internal capsule, a pathway that explained 17.13% of the total effect. Interestingly, we also identified a reverse mediation pattern where the effect of ICVF in the right cingulum hippocampus on GBM was mediated by UDP-glucuronate in brain tissue, with a mediation proportion of 8.46%.

In non-GBM, FA in the posterior limb of the left internal capsule served as a crucial mediator in the association between CSF orotate and non-GBM, contributing 17.44% to the mediation effect. Finally, ICVF in the genu of the corpus callosum was a key node linking plasma imidazole propionate with non-GBM risk, explaining 7.84% of the effect.

These findings reveal the presence of cross-omics mediating effects, elucidating how alterations in brain microstructural features and specific metabolite levels may intricately interact in glioma pathogenesis. Detailed results of the mediation analysis are provided in **Supplementary Tables 11**, **12**.

Similarly, to explore the potential mechanisms by which *HEATR3* influences glioma malignant phenotypes, we evaluated the mediating effects of IDPs in the association between *HEATR3* and glioma risk using the two-step MR framework. The results demonstrated that the impact of *HEATR3* on glioma risk is partially mediated by specific white matter microstructural imaging phenotypes. In GBM, these IDPs mediated 4.94 - 11.12% of the risk effect conferred by elevated *HEATR3* expression across different regions (**Supplementary Tables 13**, **14**). These findings suggest that *HEATR3* may exert its oncogenic effects by compromising white matter microstructural integrity, providing a potential structural basis for its biological functions.

## 4 Discussion

By constructing a comprehensive multi-omics Mendelian randomization framework that utilizes genetic variants as instrumental variables, this study systematically evaluated the causal relationships among IDPs, metabolites, regional gene expression across 13 brain regions, and the risk of overall glioma, GBM, and non-GBM. This methodological integration transcends simple association analyses, effectively mitigating confounding factors and reverse causality biases inherent in traditional observational studies. For the first time, we provide robust causal evidence regarding the influence of brain structure, metabolism, and regional gene expression on glioma risk. Through TSMR, two-step MR mediation analysis, and SMR, we systematically elucidated the causal effects of multi-omics factors—ranging from macroscopic imaging phenotypes to microscopic molecular and genetic levels—on glioma. Our findings not only highlight the universal risk status of microstructural diffusion alterations and quantify the pathways through which specific metabolites mediate glioma risk by altering white matter integrity, but also identify and experimentally validate *HEATR3*, a core oncogene exerting pan-brain causal effects. These discoveries offer critical genetic insights into neuro-oncological interactions and lay a theoretical foundation for early risk prediction and the development of novel therapeutic targets.

Although previous studies have explored the association between T1/T2-weighted MRI-derived brain phenotypes and GBM using MR methods (35), the limitations of conventional MRI in characterizing microstructures have become increasingly apparent as recent research reveals the complex interplay between glioma and white matter tracts (36). Consequently, diffusion MRI (dMRI) parameters, which can directly reflect white matter microstructural characteristics, have garnered widespread attention (37). However, causal inference studies linking dMRI parameters to glioma genetics remain scarce. Therefore, by systematically evaluating dMRI and sMRI-derived phenotypes (IDPs), this study highlights the central role of white matter microstructural changes in glioma susceptibility. Elevated intracellular volume fraction (ICVF), a marker of reduced white matter tract integrity or increased cellular density (38), is often associated with gliosis or tumor infiltration (39). Our analysis demonstrated that elevated ICVF in key associative structures, such as the genu of the corpus callosum, is significantly correlated with increased glioma risk. This finding not only aligns with the pathological characteristic of glioma cells migrating long distances along white matter tracts (40) but also explains the clinical imaging phenomenon where tumors frequently spread along “highways” such as the corpus callosum (41, 42). Thus, the genetically determined high-ICVF phenotype may represent a neural microenvironment structurally more conducive to tumor migration—that is, higher axonal density providing a more effective scaffold for tumor initiation and progression. This pre-existing state of high cellular density may also reflect a genetic predisposition to neuroinflammation and gliosis (43), thereby fostering a local milieu permissive for tumorigenesis (44). The cingulum tract is a critical pathway connecting the frontal lobe, parietal lobe, and medial temporal lobe, involved in regulating executive control, emotion, and episodic memory (45). The elevation of ICVF in the right cingulum indicates a genetic link between glioma susceptibility and compromised white matter integrity in the limbic system. This not only supports a connection between glioma pathogenesis and neural network dysfunction but also provides evidence for the impact of psychiatric disorders on glioma risk (46, 47). Contrasting the risk posed by increased ICVF, elevated mean diffusivity (MD) in the posterior limb of the right internal capsule demonstrated a protective effect across all three glioma categories. MD represents the freedom of water molecule diffusion within tissues; its increase denotes freer diffusion, generally indicating looser tissue structure (48). The protective effect of elevated MD contrasts intriguingly with the pathogenic effect of elevated ICVF, together suggesting that genetically looser white matter structures may exhibit stronger resistance to tumor progression. These results underscore the potential of diffusion tensor imaging parameters as predictive biomarkers for glioma risk and collectively reveal the universal and central role of white matter microstructure in dictating glioma susceptibility.

A pivotal finding of this study is that orotate significantly increases GBM risk consistently across three physiological compartments: plasma, CSF, and brain tissue. This cross-compartment consistency robustly reinforces orotate as a potential biomarker and therapeutic target for GBM. Orotate is a critical intermediate in the *de novo* pyrimidine synthesis pathway (49), catalyzed by dihydroorotate dehydrogenase (DHODH). This pathway is already recognized as a promising therapeutic target for glioma, with clinical trials currently evaluating DHODH inhibitors (50–52). Genetically predisposed elevations in orotate may herald the dysregulation or hyperactivation of endogenous pyrimidine synthesis via DHODH. Importantly, this metabolic dysregulation is not merely a byproduct of fueling the hyperproliferative demands of GBM; evidence suggests it actively contributes to shaping a distinct, immunosuppressive tumor microenvironment. For instance, recent studies indicate that DHODH inhibition can not only block tumor cell proliferation but also enhance the anti-tumor immune activity of natural killer (NK) cells (53). Thus, targeting this metabolic-immune axis may offer dual therapeutic benefits. This consistent cross-tissue finding suggests that orotate metabolic derangement may be a systemic genetic trait; even with the blood-brain barrier separating the peripheral and central compartments, it continues to play a critical pathogenic role in GBM either through barrier disruption or local synthetic dysregulation. KEGG pathway enrichment analysis of the identified metabolites revealed significant metabolic heterogeneity between GBM and non-GBM. GBM-specific enriched pathways predominantly converged on rapid cell proliferation and tumor microenvironment manipulation. For example, choline metabolism, a classic hallmark of malignancy, fuels accelerated membrane phospholipid synthesis (54) and is vital for GBM cell differentiation (55). Furthermore, the enrichment of necroptosis and efferocytosis hints at potential links between GBM and central nervous system programmed cell death regulatory mechanisms (56). Conversely, non-GBM specifically enriched for multiple amino acid metabolism pathways and central carbon metabolism. Non-GBM may rely more heavily on core carbon sources and branched-chain amino acids as energy substrates or signaling molecules to support a relatively slower, yet highly adaptive, growth pattern. These diverging metabolic profiles provide a genetic rationale for developing customized, metabolomics-based targeted interventions for different glioma grades.

Mediation analysis uncovered how metabolic stress might project onto specific white matter tracts. For instance, the causal effect of plasma orotate on overall glioma risk was partially mediated by the ICVF in the right cingulum hippocampus. We hypothesize that the high metabolic stress indicated by genetically elevated orotate levels may early on disrupt cellular homeostasis in these highly active regions, leading to white matter microstructural alterations that subsequently provide opportunities for tumor initiation and dissemination. This study also uncovered a reverse mediation pattern: the effect of ICVF in the right cingulum hippocampus on GBM was partially mediated by UDP-glucuronate in brain tissue. UDP-glucuronate is a precursor for extracellular matrix glycosaminoglycans, which are implicated in the progression of various tumors, including GBM (57). This reverse causal chain suggests that the genetic integrity of brain white matter microstructure may dictate the homeostasis and clearance capacity of local brain metabolites (58). Structural defects could lead to the abnormal accumulation of metabolites in brain tissue, offering metabolic support for tumors or triggering sustained neuroinflammation, thereby indirectly increasing GBM susceptibility. Notably, plasma imidazole propionate [a known gut microbiota-derived metabolite (59)] mediated non-GBM risk via the ICVF in the genu of the corpus callosum. This implies that microbial products may influence the microstructural integrity of central white matter via the gut-brain axis, thereby modulating glioma oncogenesis.

SMR analysis based on *cis*-eQTL data from 13 brain regions revealed that specific areas, such as the cortex, are significantly enriched for genes related to DNA replication and cell cycle regulation. This association not only suggests that brain regions with active DNA replication may be more prone to becoming glioma initiation “hotspots,” but it also aligns with the hallmark of high replication stress in gliomas (60, 61). A previous large-scale GWAS meta-analysis identified the 16q12.1 chromosomal region, where *HEATR3* is located, as a significant susceptibility locus for GBM (62). The consistency underscores the accuracy and reliability of our analysis. Our study consolidating this genetic association through two independent and complementary analytical methods. Through this approach, we successfully confirmed the robust, pan-regional pathogenic role of *HEATR3* expression across all 13 brain regions evaluated. The *HEATR3* protein is known to participate in ribosome biogenesis and ribosomal protein transport (63) and has been implicated in breast cancer progression (64). Ribosome biogenesis is a core process mandatory for rapid proliferation in all cells and is involved in the initiation and progression of multiple tumors, including glioma (65, 66). Genetically determined elevations in *HEATR3* expression may universally lower the threshold for glioma genesis by enhancing translational efficiency and the proliferative potential of glial cells. Further clinical validations and functional experiments provided compelling evidence supporting this mechanism. Collectively, these results establish *HEATR3* as a critical oncogene in glioma, endowing it with the potential to serve as a prognostic biomarker and therapeutic target.

While the aforementioned results provide robust causal evidence for glioma pathogenesis, this study possesses certain limitations. First, the GWAS summary data utilized were primarily derived from European populations, limiting the generalizability of the findings to other ethnic groups; future studies urgently need to incorporate more diverse, non-European population data to confirm the universality of these causal links. Second, although MR analyses offered causal evidence and mediation pathways, the underlying biological mechanisms require further *in vivo* functional validation. Additionally, despite sensitivity analyses ruling out significant pleiotropy, MR cannot entirely eliminate the influence of unknown horizontal pleiotropy. Finally, while the role of *HEATR3* in driving malignant progression has been confirmed at the cellular level, its *in vivo* functions remain to be validated through animal models.

## 5 Conclusion

From a genetic perspective, this study reveals the causal roles of brain microstructural abnormalities, metabolic dysregulation, and region-specific gene expression in the development and progression of glioma. It provides novel multi-layered evidence regarding glioma pathogenesis and lays a theoretical foundation for the future development of early risk prediction strategies and novel therapeutic targets based on imaging and metabolomics.

## Data Availability

The datasets presented in this study can be found in online repositories. The names of the repository/repositories and accession number(s) can be found in the article/**Supplementary Material**.
