# Supplementary material for: Imaging-mediated genetic effects link brain microstructure, metabolic profiles, and regional transcription to glioma susceptibility
Source: Front Immunol. 2026 Jul 3;17:1870121. doi: 10.3389/fimmu.2026.1870121 (PMC13376868; doi:10.3389/fimmu.2026.1870121)
Supplement: Supplementary file 1 [file Image1.pdf]

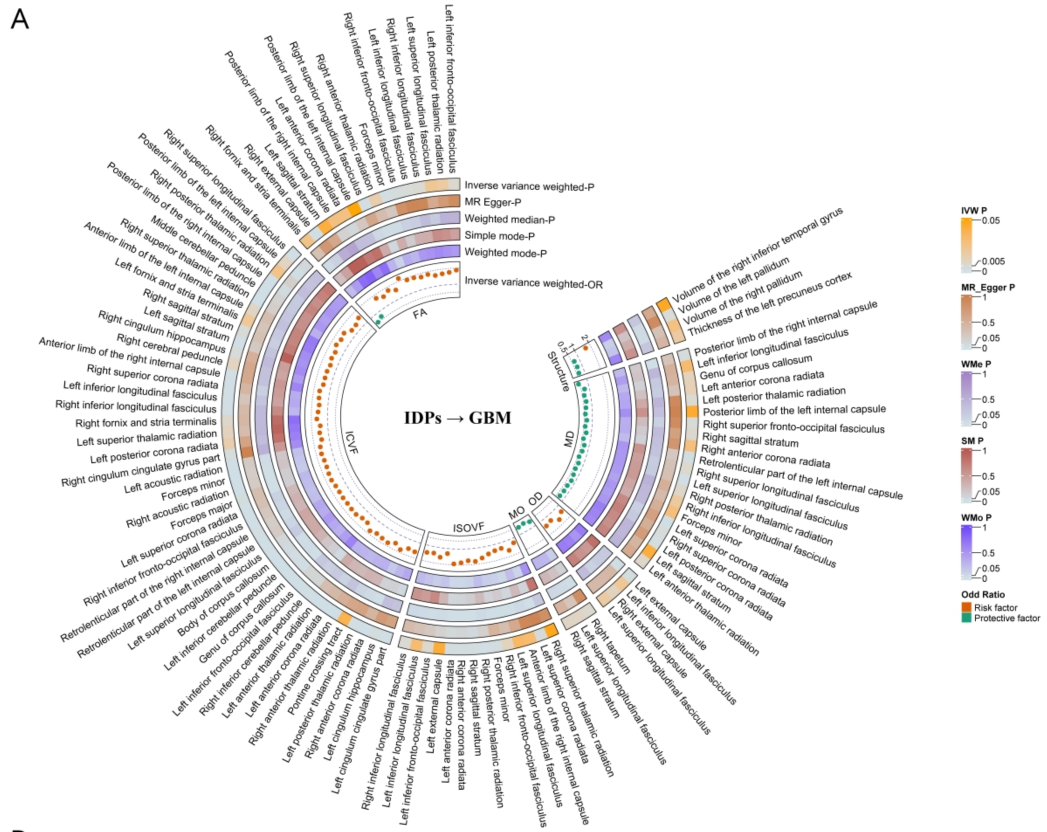

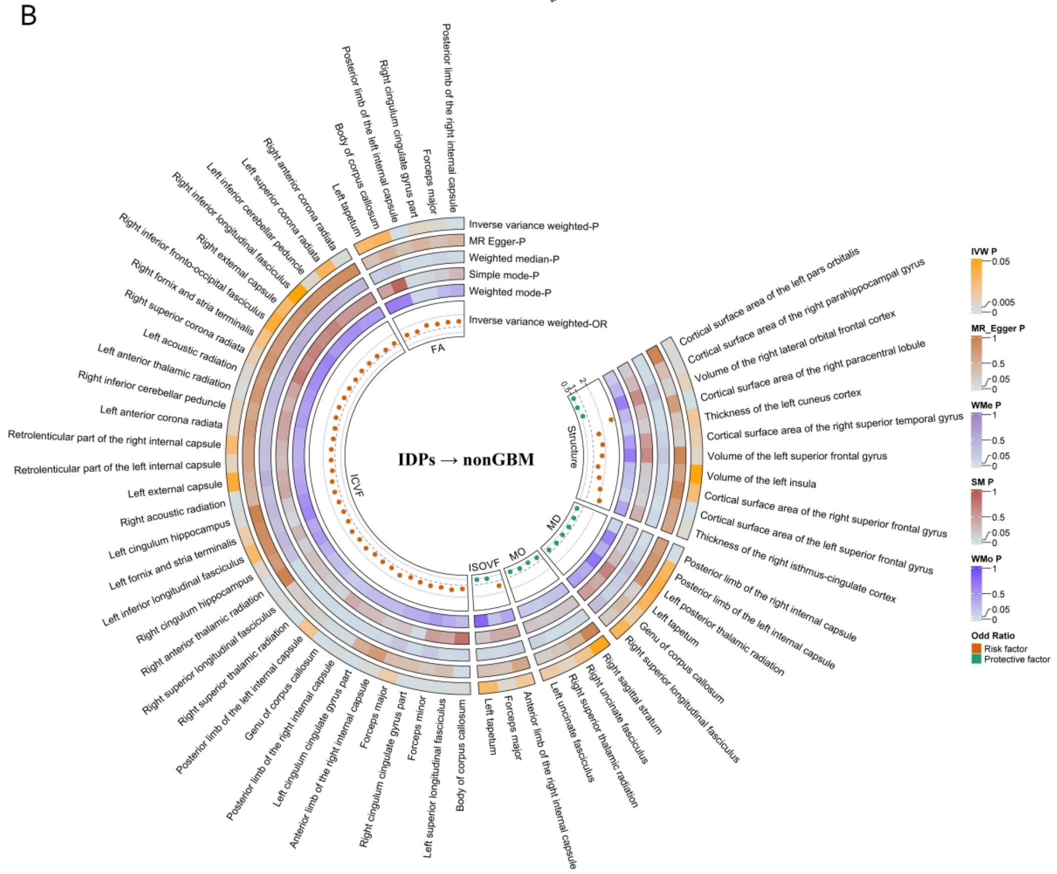

Figure S1. Mendelian randomization analysis of causal effects of IDPs on GBM and nonGBM.

(A). Circular heatmap showing the causal effects of IDPs on GBM.

(B). Circular heatmap showing the causal effects of IDPs on nonGBM.

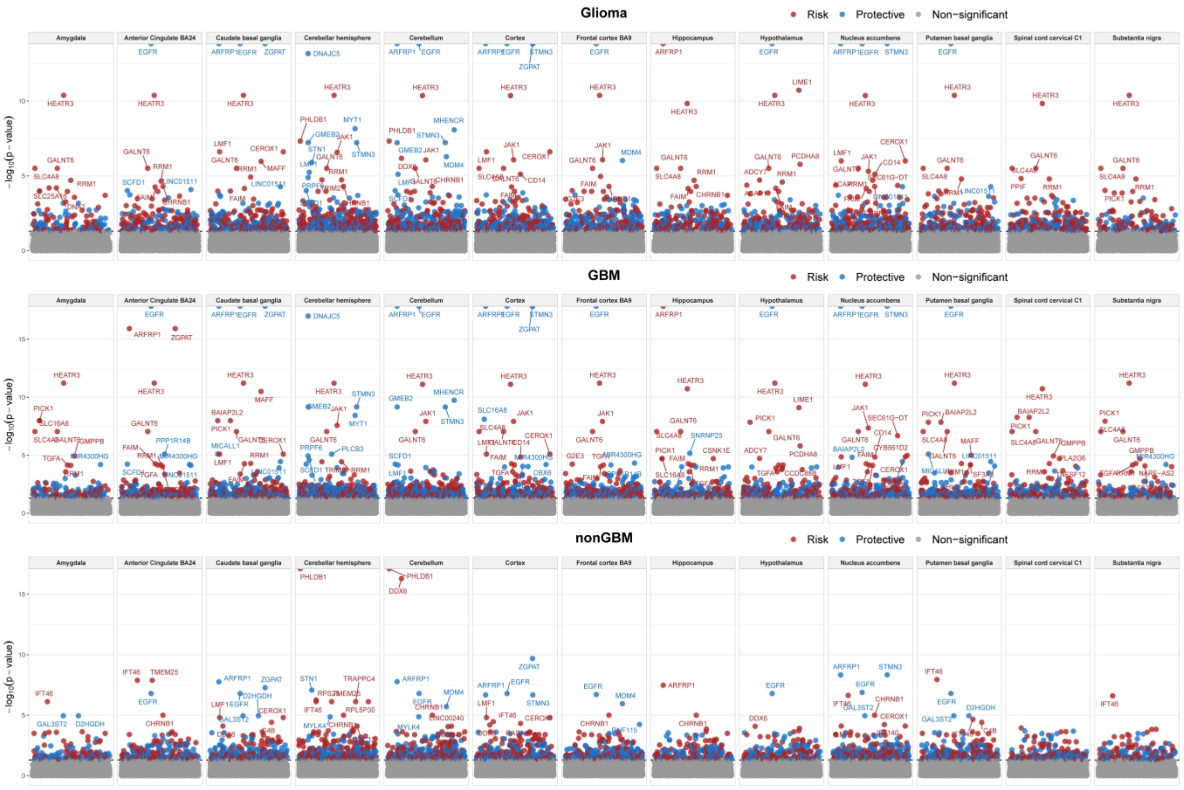

Figure S2. Manhattan plot of TSMR results for gene expression in 13 brain regions across glioma, GBM and nonGBM.

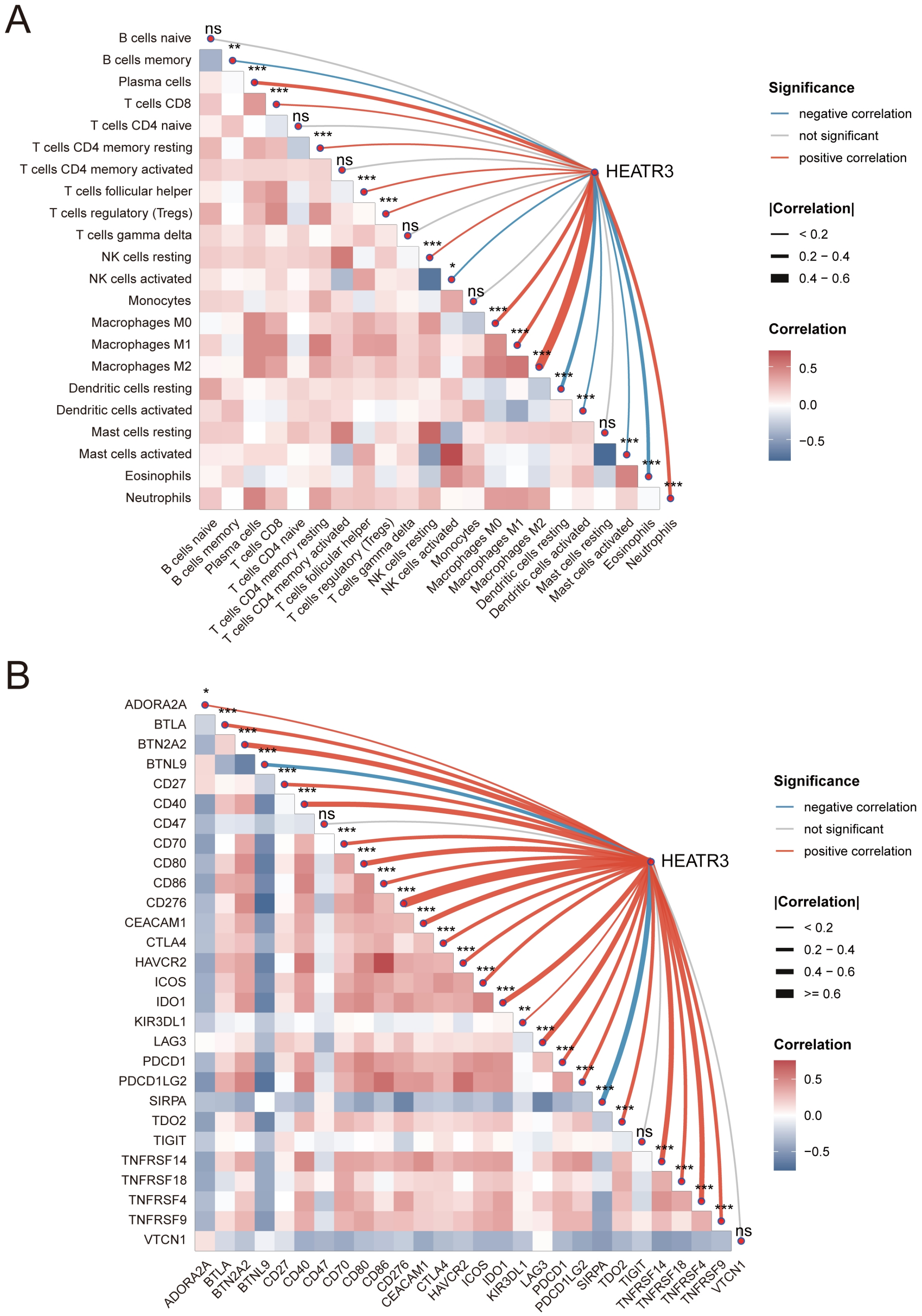

Figure S3. *HEATR3* expression reflects the immune characteristics of glioma.
(A). Correlation between *HEATR3* expression and immune cell infiltration levels in glioma.
(B). Correlation between *HEATR3* expression and immune checkpoint expression in glioma.

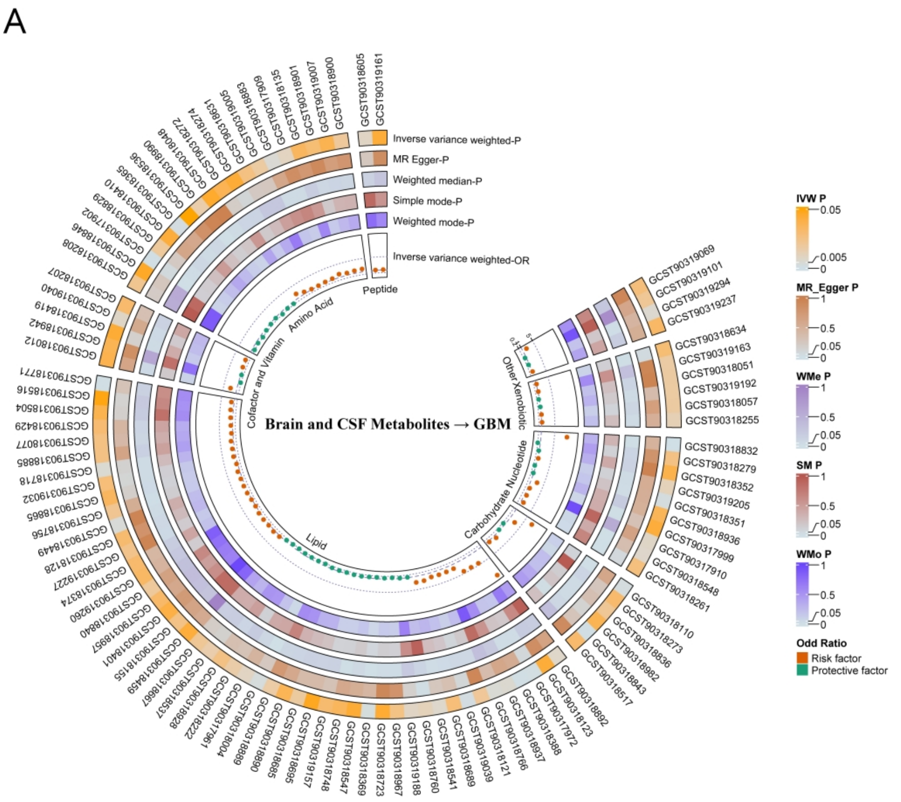

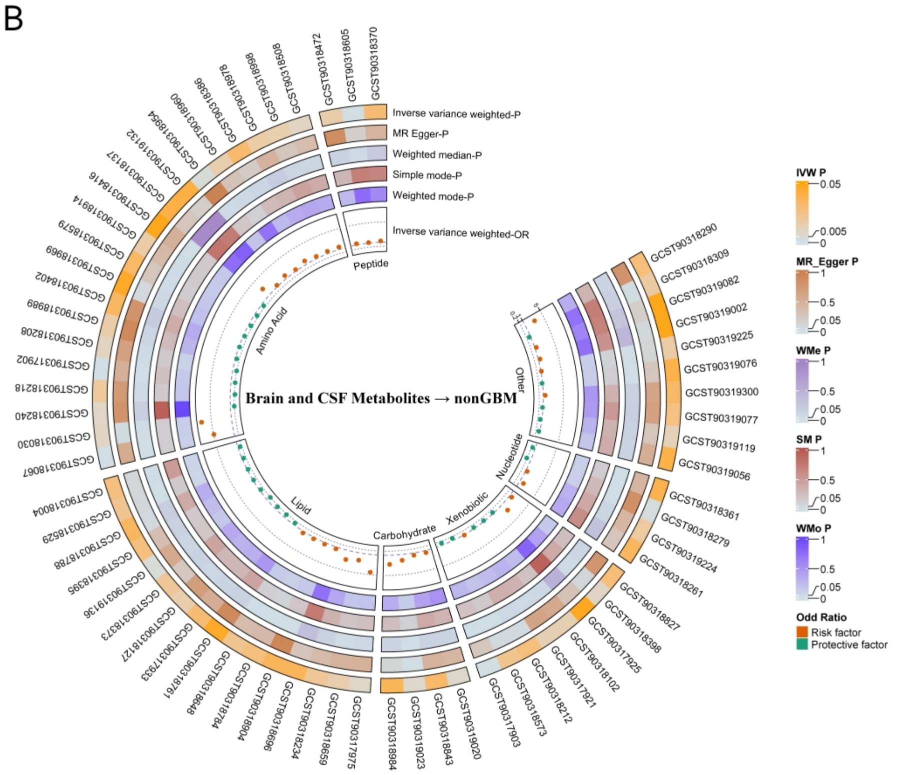

Figure S4. Mendelian randomization analysis of causal effects of brain and CSF metabolites on GBM and nonGBM.

(A). Circular heatmap showing the causal effects of brain and CSF metabolites on GBM.

(B). Circular heatmap showing the causal effects of brain and CSF metabolites on nonGBM.

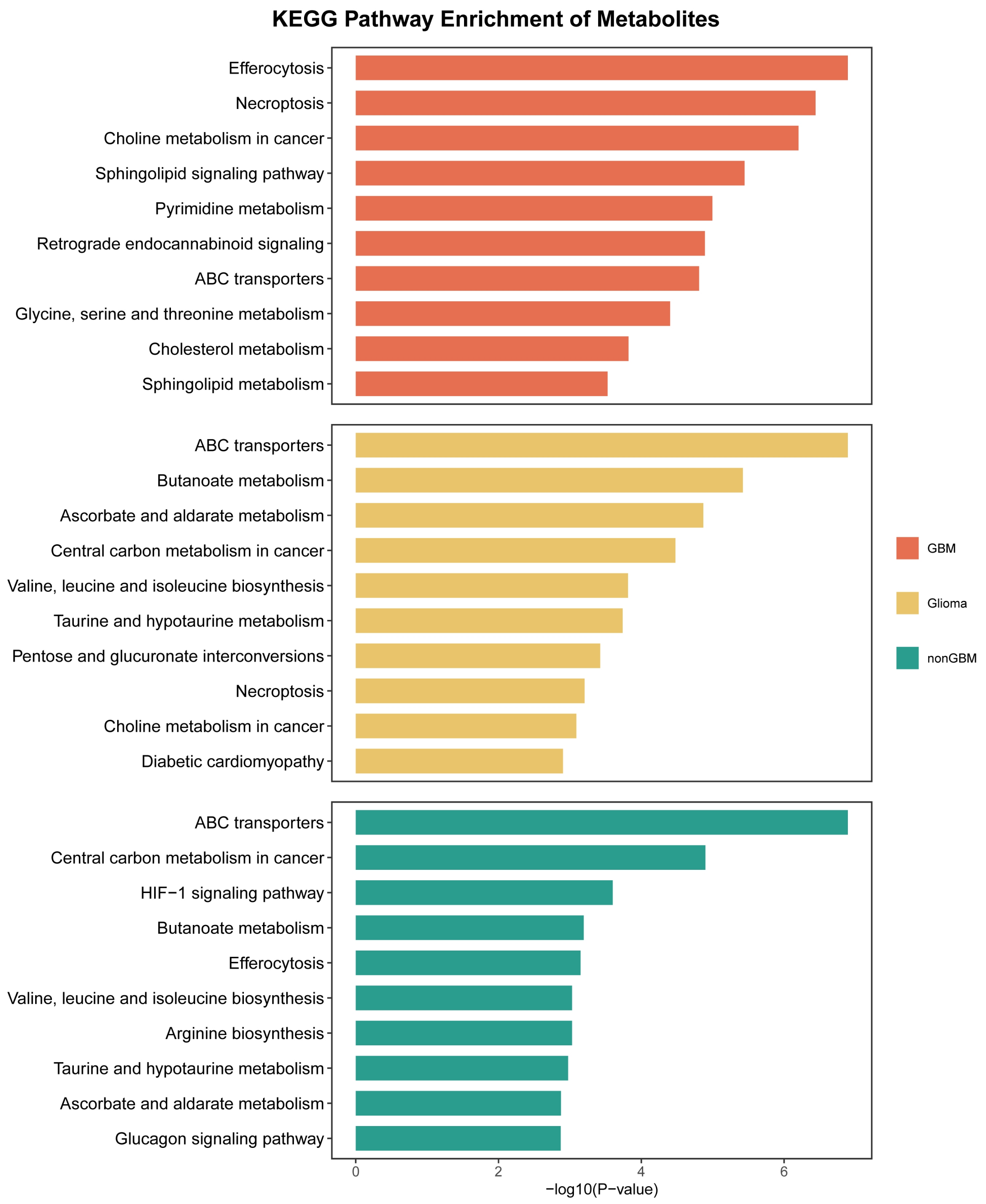

Figure S5. KEGG enrichment analysis of metabolites with causal effects on overall glioma, GBM, and nonGBM.

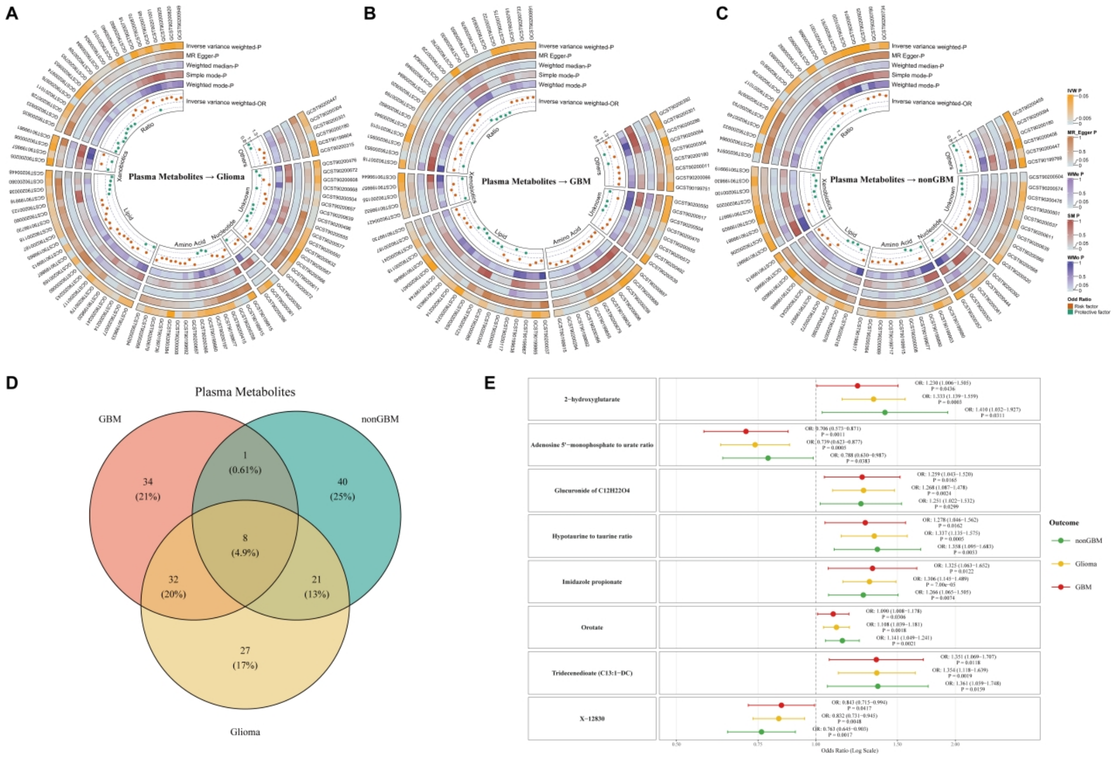

Figure S6. Mendelian randomization analysis of the causal effects of plasma metabolites on glioma.

(A).Circular heatmap showing the causal effects of plasma metabolites on overall glioma. (B).Circular heatmap showing the causal effects of plasma metabolites on GBM. (C).Circular heatmap showing the causal effects of plasma metabolites on nonGBM. (D).Venn diagram illustrating plasma metabolites with significant causal effects on overall glioma, GBM, and nonGBM. (E).Forest plot depicting the causal effects of plasma metabolites significant for overall glioma, GBM, and nonGBM.
