# Supplementary material for: Imaging-mediated genetic effects link brain microstructure, metabolic profiles, and regional transcription to glioma susceptibility
Source: Front Immunol. 2026 Jul 3;17:1870121. doi: 10.3389/fimmu.2026.1870121 (PMC13376868; doi:10.3389/fimmu.2026.1870121)
Supplement: Supplementary file 2 [file Presentation1.pdf]

Cell Experiments

Cell Culture

The human glioma cell line U251 was purchased from Saili Biotechnology (Shanghai, China) and cultured in DMEM medium supplemented with 10% fetal bovine serum and 1% penicillin-streptomycin in a 37°C, 5% CO_2 incubator.

siRNA Transfection

siRNAs targeting *HEATR3* were obtained from Sangon Biotech (Shanghai, China) with the sequence: GAGCAGUGUCUAUAUUCAA/dT//dT/;GGCACAAUUUGGAAUCUAA/dT//dT/;GCAGUUGACCUAUGUUCUA/dT//dT/. Transfection was performed using Lipofectamine® 3000 according to the manufacturer's instructions. The knockdown efficiency of *HEATR3* at the mRNA and protein levels was evaluated by RT-qPCR and Western blot. Primers were purchased from Sunya Biotech (Zhejiang, China) with the sequence:CGGCTCTCACCAACTACCTC (Forward), TCCAGGAGGGACACAAGACT (Reverse) . For western blotting, the primary antibody anti-HEATR3 (Wuhan Sanying, Cat No. 27335-1-AP) used at a dilution of 1:1000.

Cell Proliferation Assay

Cell proliferation was evaluated using the CCK-8 assay(Beyotime, China, No. C0037). Transfected cells were seeded in 96-well plates at a density of 1,000 cells per well. On days 1, 2, 3, and 4 post-seeding, 10 μL of CCK-8 reagent was added to each well. After a 2-hour incubation at 37°C, the absorbance at 450 nm (OD450) was measured using a microplate reader .

Cell Migration Assay

Cell migration was assessed using Transwell chambers. Serum-free cell suspensions (5×10^4 cells/mL) were added to the upper chamber, while medium containing 10% FBS was added to the lower chamber as a chemoattractant. After 24 hours of incubation, non-migrating cells in the upper chamber were removed with a cotton swab. Cells that migrated to the lower chamber were fixed with 4% paraformaldehyde, stained with 0.1% crystal violet, and counted under a microscope in five randomly selected fields.

Wound Healing Assay

Cells were seeded at an appropriate density in 6-well plates. Upon reaching approximately 90% confluence, an artificial scratch was made using a sterile 1000 μL pipette tip. After washing with PBS to remove floating cells, the medium was replaced with DMEM containing 1% FBS. Images were captured at the same position under a microscope at 0, 12, and 24 hours post-scratching. Scratch widths were measured using ImageJ software to calculate the migration distance.

Colony Formation Assay

Transfected cells were seeded at 1,000 cells/well in 6-well plates and cultured routinely for 10 days. Once macroscopic colonies formed, the medium was discarded, and cells were washed with PBS, fixed with 4% paraformaldehyde, and stained with 0.1% crystal violet. Colonies containing >50 cells were counted.

Cell Apoptosis Assay

Apoptosis was detected using the Annexin V-FITC dual-staining kit (Beyotime, China, No. C1062S). After washing with pre-chilled PBS, cells were resuspended in binding buffer according to the manufacturer's instructions, stained with Annexin V-FITC and PI, and incubated in the dark at room temperature for 30 minutes. The proportions of early and late apoptotic cells were immediately analyzed via flow cytometry.

Statistical Analysis

All *in vitro* experiments were independently repeated at least three times. Data were presented as mean ± standard deviation. Comparisons between two groups were performed using Student’s t-test, while multi-group comparisons were analyzed using one-way ANOVA. Statistical analysis and graphing were performed using GraphPad Prism 5 software. $P < 0.05$ was considered statistically significant.
