# Supplementary figures and images for: Imaging-mediated genetic effects link brain microstructure, metabolic profiles, and regional transcription to glioma susceptibility
Source: Front Immunol. 2026 Jul 3;17:1870121. doi: 10.3389/fimmu.2026.1870121 (PMC13376868; doi:10.3389/fimmu.2026.1870121)

The full-length Western blot membrane image of Figure 5D

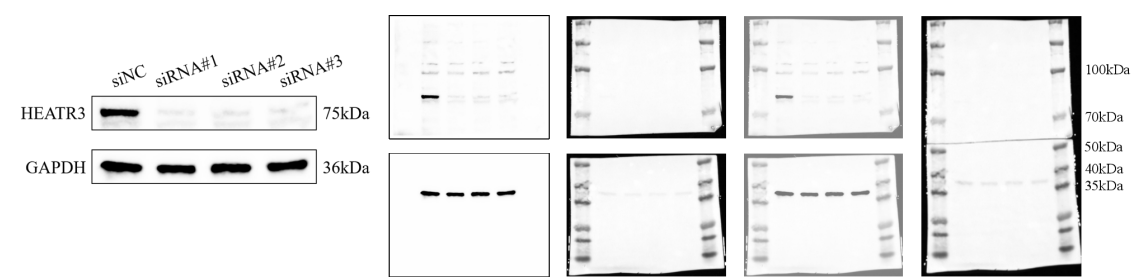

Supplement: Supplementary file 3 [file Presentation2.pdf]
