# Supplementary material for: Imaging-mediated genetic effects link brain microstructure, metabolic profiles, and regional transcription to glioma susceptibility
Source: Front Immunol. 2026 Jul 3;17:1870121. doi: 10.3389/fimmu.2026.1870121 (PMC13376868; doi:10.3389/fimmu.2026.1870121)
Supplement: Supplementary file 4 [file Presentation3.pdf]

SMR results for the remaining 12 brain regions

In the amygdala, we identified 10 genes associated with glioma and its subtypes, including 7 risk-increasing genes and 3 protective genes (Figure 1).

In the anterior cingulate cortex, we identified 11 associated genes, including 6 risk-increasing and 5 protective genes (Figure 2).

In the caudate, we identified 10 associated genes, including 5 risk-increasing and 5 protective genes (Figure 3).

In the cerebellar hemisphere, we identified 10 associated genes, including 6 risk-increasing and 4 protective genes (Figure 4).

In the cerebellum, we identified 17 associated genes, including 10 risk-increasing and 7 protective genes (Figure 5).

In the frontal cortex, we identified 10 associated genes, including 7 risk-increasing and 3 protective genes (Figure 6).

In the hippocampus, we identified 6 associated genes, including 5 risk-increasing and 1 protective gene (Figure 7).

In the hypothalamus, we identified 8 associated genes, including 6 risk-increasing and 2 protective genes (Figure 8).

In the nucleus accumbens, we identified 9 associated genes, including 7 risk-increasing and 2 protective genes (Figure 9).

In the putamen, we identified 8 associated genes, including 4 risk-increasing and 4 protective genes (Figure 10).

In the cervical spinal cord (C-1), we identified 6 associated genes, including 5 risk-increasing and 1 protective gene (Figure 11).

In the substantia nigra, we identified 9 associated genes, including 7 risk-increasing and 2 protective genes (Figure 12).

Figure 1

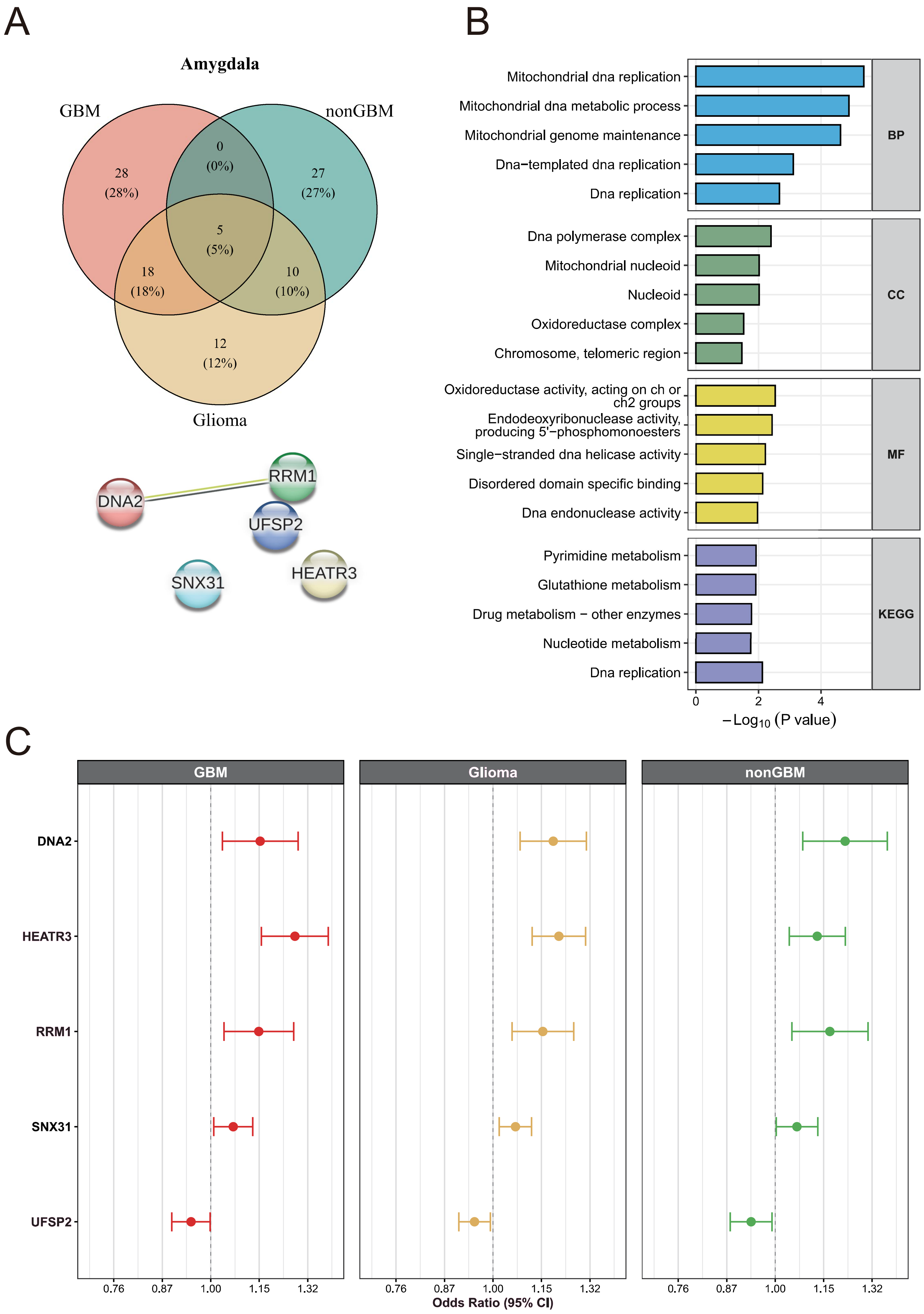

A
Amygdala
GBM
nonGBM
Glioma
28
(28%)
0
(0%)
27
(27%)
5
(5%)
18
(18%)
10
(10%)
12
(12%)
DNA2
RRM1
UFSP2
SNX31
HEATR3
B
Mitochondrial dna replication
Mitochondrial dna metabolic process
Mitochondrial genome maintenance
Dna−templated dna replication
Dna replication
BP
Dna polymerase complex
Mitochondrial nucleoid
Nucleoid
Oxidoreductase complex
Chromosome, telomeric region
CC
Oxidoreductase activity, acting on ch or ch2 groups
Endodeoxyribonuclease activity, producing 5'−phosphomonoesters
Single−stranded dna helicase activity
Disordered domain specific binding
Dna endonuclease activity
MF
Pyrimidine metabolism
Glutathione metabolism
Drug metabolism − other enzymes
Nucleotide metabolism
Dna replication
KEGG
0
2
4
−Log10 (P value)
C
GBM
Glioma
nonGBM
DNA2
HEATR3
RRM1
SNX31
UFSP2
0.76
0.87
1.00
1.15
1.32
Odds Ratio (95% CI)

Figure 2

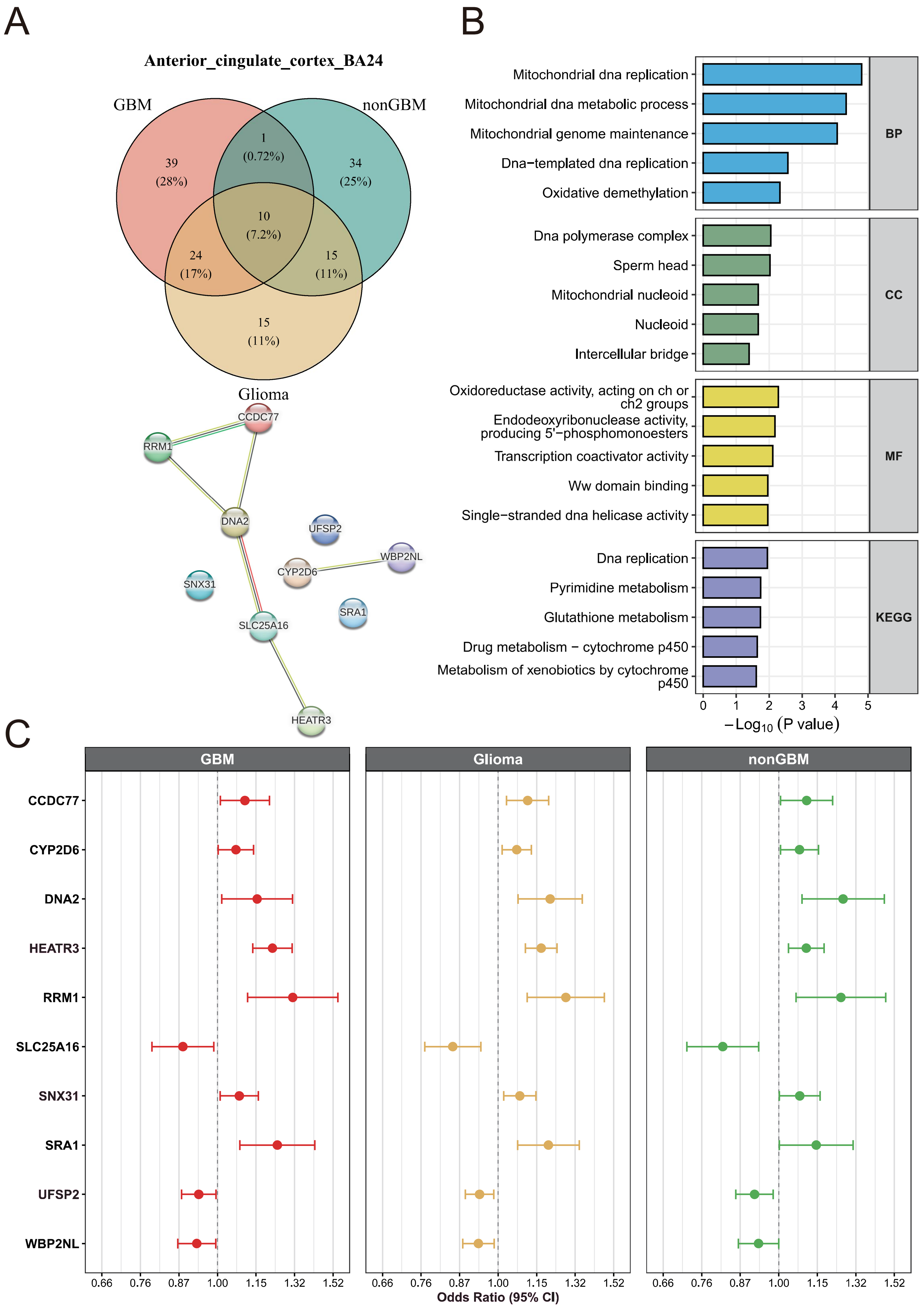
A
Anterior_cingulate_cortex_BA24
GBM
nonGBM
Glioma
39
(28%)
1
(0.72%)
34
(25%)
10
(7.2%)
24
(17%)
15
(11%)
15
(11%)
CCDC77
RRM1
DNA2
UFSP2
WBP2NL
CYP2D6
SNX31
SRA1
SLC25A16
HEATR3
B
Mitochondrial dna replication
Mitochondrial dna metabolic process
Mitochondrial genome maintenance
Dna−templated dna replication
Oxidative demethylation
BP
Dna polymerase complex
Sperm head
Mitochondrial nucleoid
Nucleoid
Intercellular bridge
CC
Oxidoreductase activity, acting on ch or ch2 groups
Endodeoxyribonuclease activity, producing 5'−phosphomonoesters
Transcription coactivator activity
Ww domain binding
Single−stranded dna helicase activity
MF
Dna replication
Pyrimidine metabolism
Glutathione metabolism
Drug metabolism − cytochrome p450
Metabolism of xenobiotics by cytochrome p450
KEGG
0 1 2 3 4 5
−Log10 (P value)
C
GBM
Glioma
nonGBM
CCDC77
CYP2D6
DNA2
HEATR3
RRM1
SLC25A16
SNX31
SRA1
UFSP2
WBP2NL
0.66 0.76 0.87 1.00 1.15 1.32 1.52
Odds Ratio (95% CI)

Figure 3

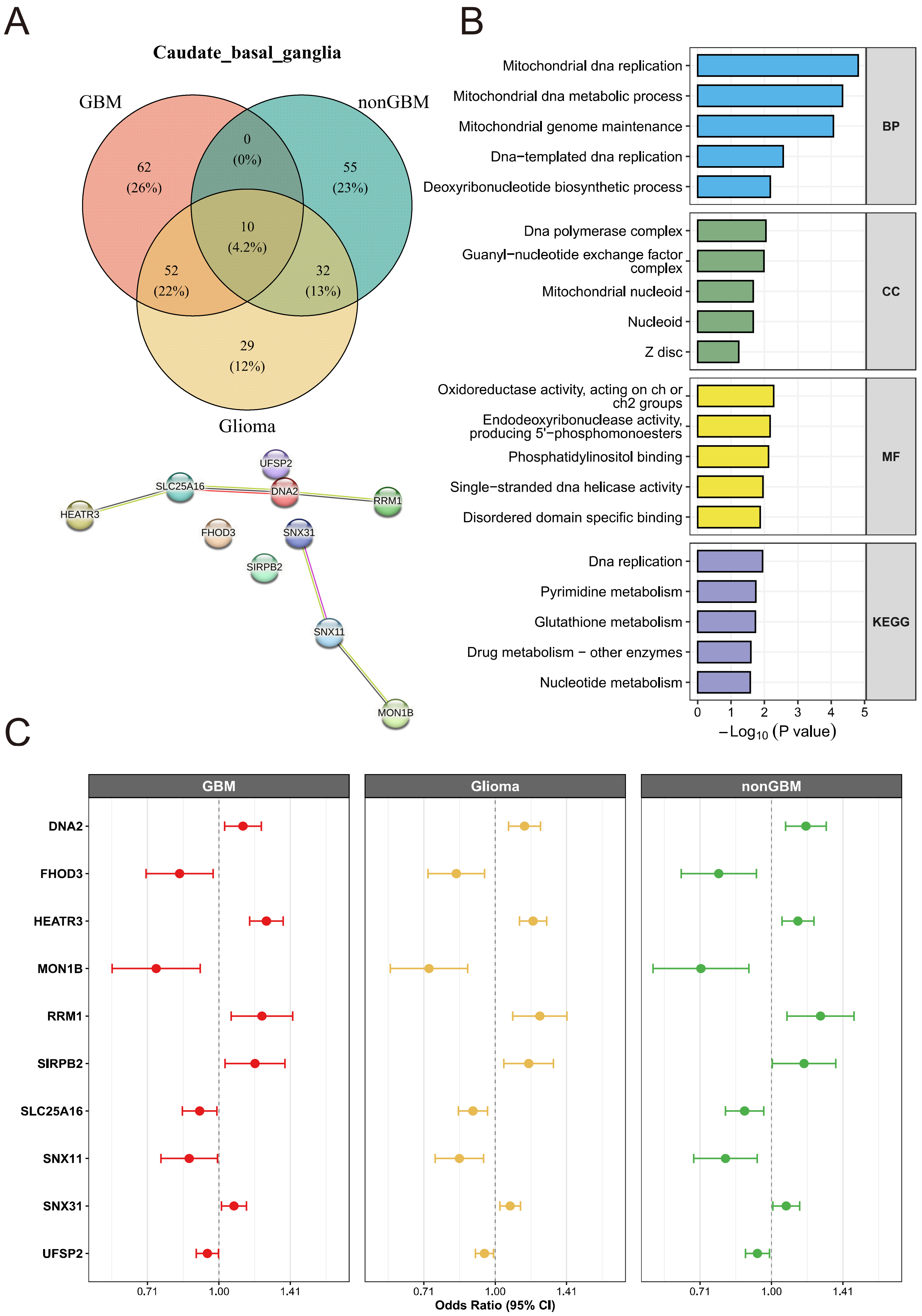
A
Caudate_basal_ganglia
GBM
nonGBM
Glioma
62
(26%)
0
(0%)
55
(23%)
10
(4.2%)
52
(22%)
32
(13%)
29
(12%)
UFSP2
SLC25A16
DNA2
RRM1
HEATR3
FHOD3
SNX31
SIRPB2
SNX11
MON1B
B
Mitochondrial dna replication
Mitochondrial dna metabolic process
Mitochondrial genome maintenance
Dna−templated dna replication
Deoxyribonucleotide biosynthetic process
BP
Dna polymerase complex
Guanyl−nucleotide exchange factor complex
Mitochondrial nucleoid
Nucleoid
Z disc
CC
Oxidoreductase activity, acting on ch or ch2 groups
Endodeoxyribonuclease activity, producing 5'−phosphomonoesters
Phosphatidylinositol binding
Single−stranded dna helicase activity
Disordered domain specific binding
MF
Dna replication
Pyrimidine metabolism
Glutathione metabolism
Drug metabolism − other enzymes
Nucleotide metabolism
KEGG
0 1 2 3 4 5
−Log10 (P value)
C
GBM
Glioma
nonGBM
DNA2
FHOD3
HEATR3
MON1B
RRM1
SIRPB2
SLC25A16
SNX11
SNX31
UFSP2
0.71 1.00 1.41
Odds Ratio (95% CI)

Figure 4

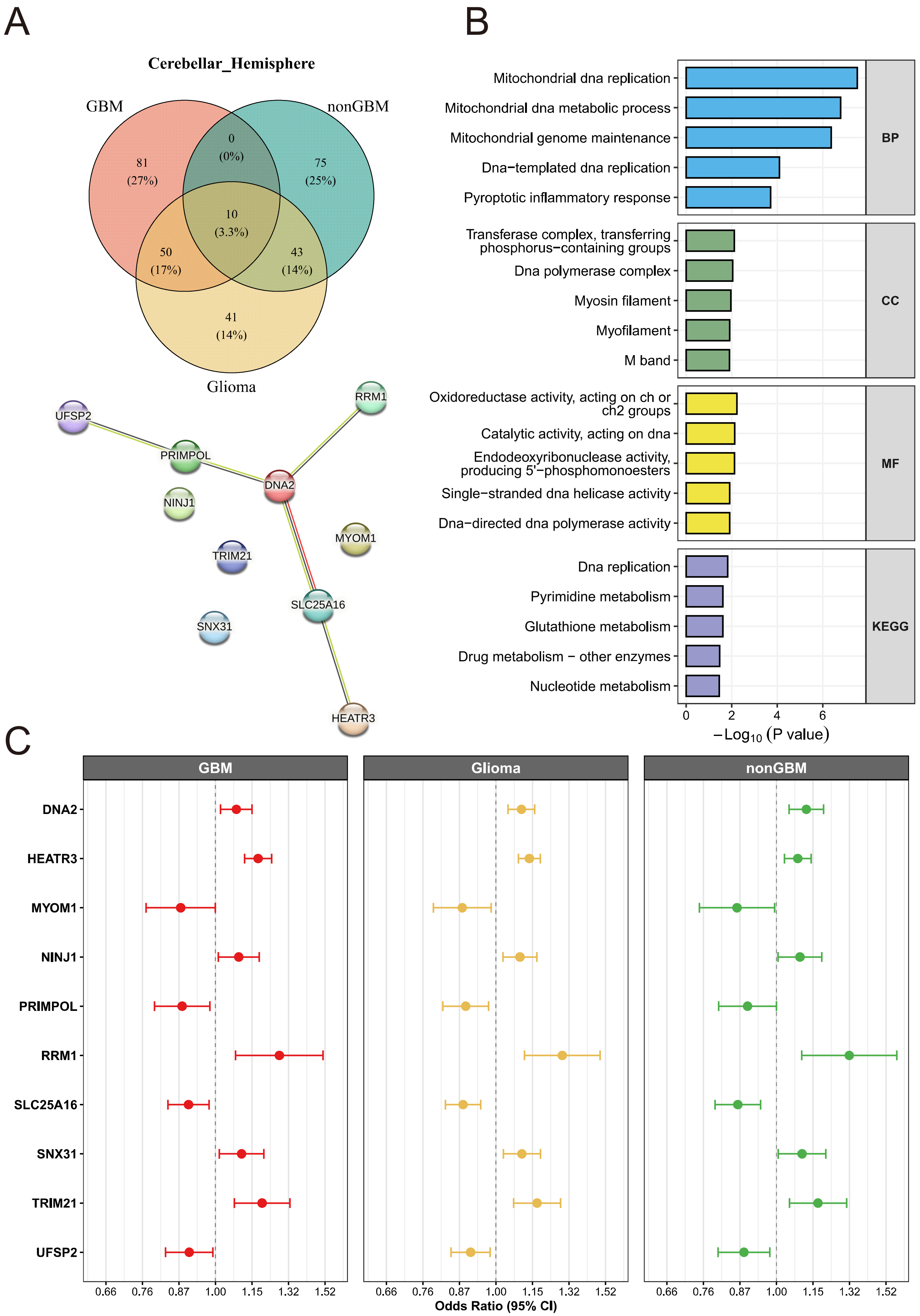
A
Cerebellar_Hemisphere
GBM
nonGBM
Glioma
81 (27%)
0 (0%)
75 (25%)
10 (3.3%)
50 (17%)
43 (14%)
41 (14%)
UFSP2
PRIMPOL
RRM1
DNA2
NINJ1
MYOM1
TRIM21
SLC25A16
SNX31
HEATR3
B
Mitochondrial dna replication
Mitochondrial dna metabolic process
Mitochondrial genome maintenance
Dna−templated dna replication
Pyroptotic inflammatory response
BP
Transferase complex, transferring phosphorus−containing groups
Dna polymerase complex
Myosin filament
Myofilament
M band
CC
Oxidoreductase activity, acting on ch or ch2 groups
Catalytic activity, acting on dna
Endodeoxyribonuclease activity, producing 5'−phosphomonoesters
Single−stranded dna helicase activity
Dna−directed dna polymerase activity
MF
Dna replication
Pyrimidine metabolism
Glutathione metabolism
Drug metabolism − other enzymes
Nucleotide metabolism
KEGG
0 2 4 6
−Log10 (P value)
C
GBM
Glioma
nonGBM
DNA2
HEATR3
MYOM1
NINJ1
PRIMPOL
RRM1
SLC25A16
SNX31
TRIM21
UFSP2
0.66 0.76 0.87 1.00 1.15 1.32 1.52
Odds Ratio (95% CI)

Figure 5

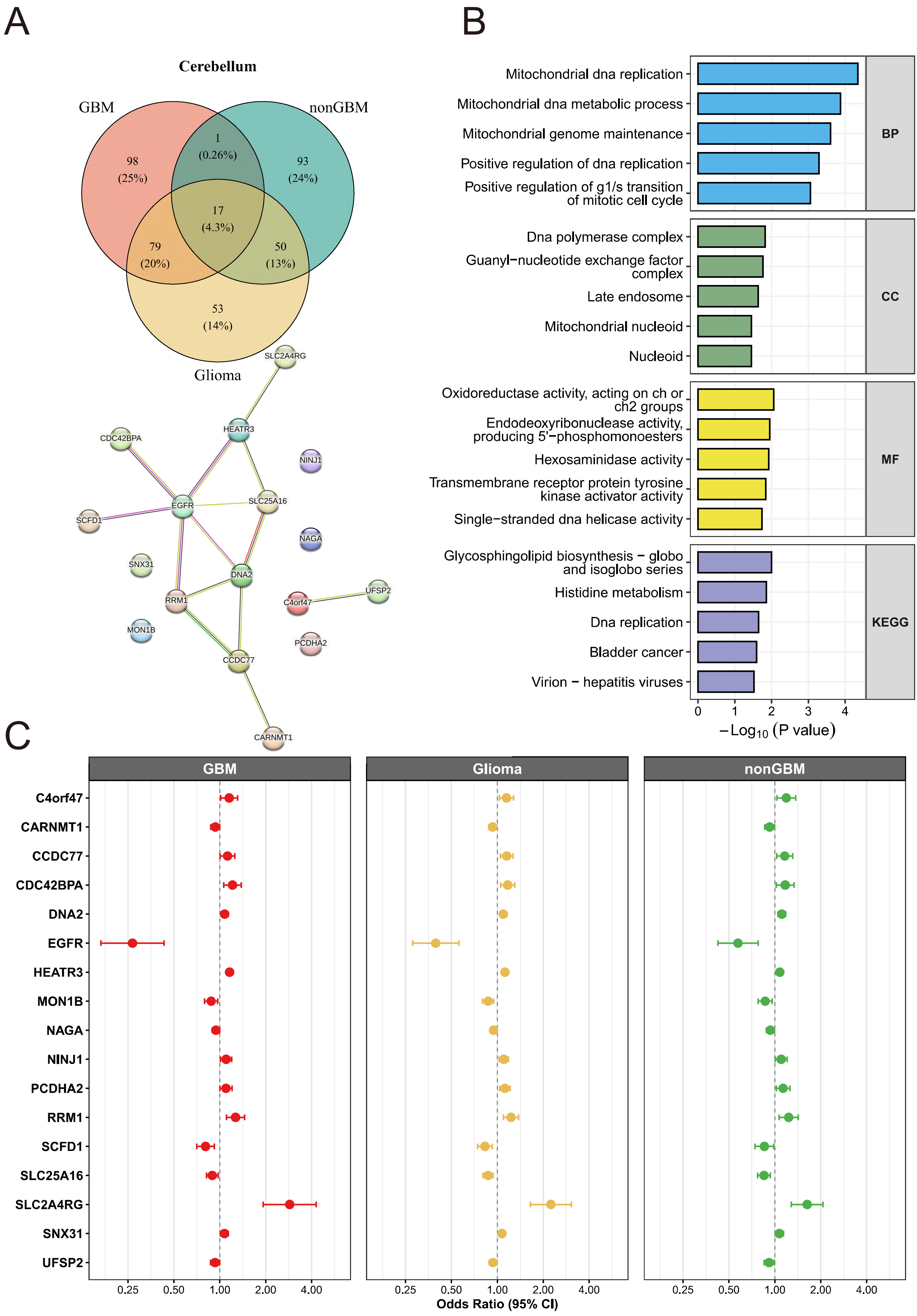
A
Cerebellum
GBM
nonGBM
Glioma
98 (25%)
1 (0.26%)
93 (24%)
17 (4.3%)
79 (20%)
50 (13%)
53 (14%)
SLC2A4RG
HEATR3
CDC42BPA
NINJ1
EGFR
SLC25A16
SCFD1
NAGA
SNX31
DNA2
UFSP2
RRM1
C4orf47
MON1B
PCDHA2
CCDC77
CARNMT1
B
Mitochondrial dna replication
Mitochondrial dna metabolic process
Mitochondrial genome maintenance
Positive regulation of dna replication
Positive regulation of g1/s transition of mitotic cell cycle
BP
Dna polymerase complex
Guanyl−nucleotide exchange factor complex
Late endosome
Mitochondrial nucleoid
Nucleoid
CC
Oxidoreductase activity, acting on ch or ch2 groups
Endodeoxyribonuclease activity, producing 5'−phosphomonoesters
Hexosaminidase activity
Transmembrane receptor protein tyrosine kinase activator activity
Single−stranded dna helicase activity
MF
Glycosphingolipid biosynthesis − globo and isoglobo series
Histidine metabolism
Dna replication
Bladder cancer
Virion − hepatitis viruses
KEGG
0 1 2 3 4
−Log10 (P value)
C
GBM
Glioma
nonGBM
C4orf47
CARNMT1
CCDC77
CDC42BPA
DNA2
EGFR
HEATR3
MON1B
NAGA
NINJ1
PCDHA2
RRM1
SCFD1
SLC25A16
SLC2A4RG
SNX31
UFSP2
0.25 0.50 1.00 2.00 4.00
Odds Ratio (95% CI)

Figure 6

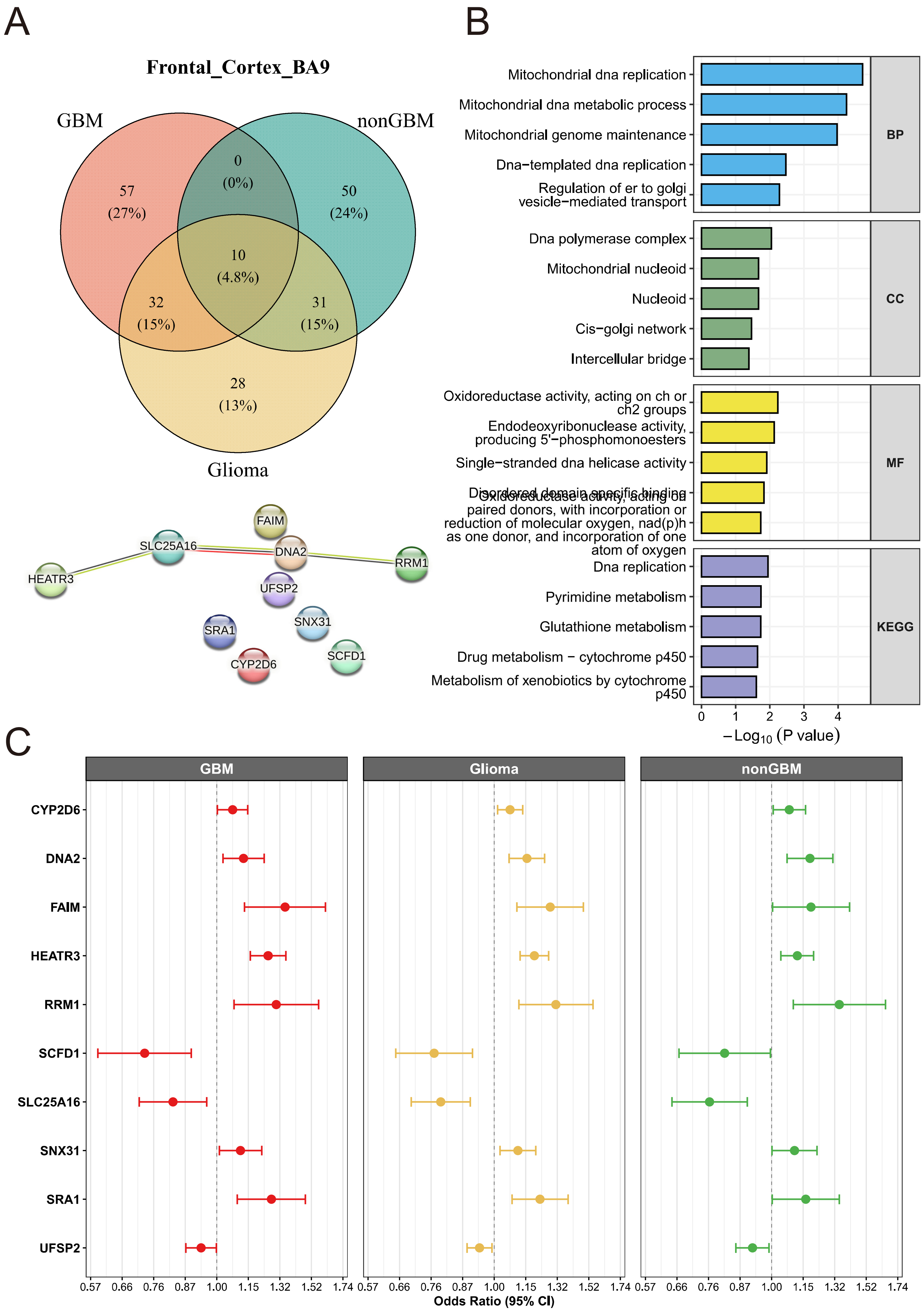

A
Frontal_Cortex_BA9
GBM
nonGBM
Glioma
57 (27%)
0 (0%)
50 (24%)
10 (4.8%)
32 (15%)
31 (15%)
28 (13%)
FAIM
SLC25A16
DNA2
RRM1
HEATR3
UFSP2
SNX31
SRA1
SCFD1
CYP2D6
B
Mitochondrial dna replication
Mitochondrial dna metabolic process
Mitochondrial genome maintenance
Dna−templated dna replication
Regulation of er to golgi vesicle−mediated transport
BP
Dna polymerase complex
Mitochondrial nucleoid
Nucleoid
Cis−golgi network
Intercellular bridge
CC
Oxidoreductase activity, acting on ch or ch2 groups
Endodeoxyribonuclease activity, producing 5'−phosphomonoesters
Single−stranded dna helicase activity
Disordered domain specific binding
Oxidoreductase activity, acting on paired donors, with incorporation or reduction of molecular oxygen, nad(p)h as one donor, and incorporation of one atom of oxygen
MF
Dna replication
Pyrimidine metabolism
Glutathione metabolism
Drug metabolism − cytochrome p450
Metabolism of xenobiotics by cytochrome p450
KEGG
0 1 2 3 4
−Log10 (P value)
C
GBM
Glioma
nonGBM
CYP2D6
DNA2
FAIM
HEATR3
RRM1
SCFD1
SLC25A16
SNX31
SRA1
UFSP2
0.57 0.66 0.76 0.87 1.00 1.15 1.32 1.52 1.74
Odds Ratio (95% CI)

Figure 7

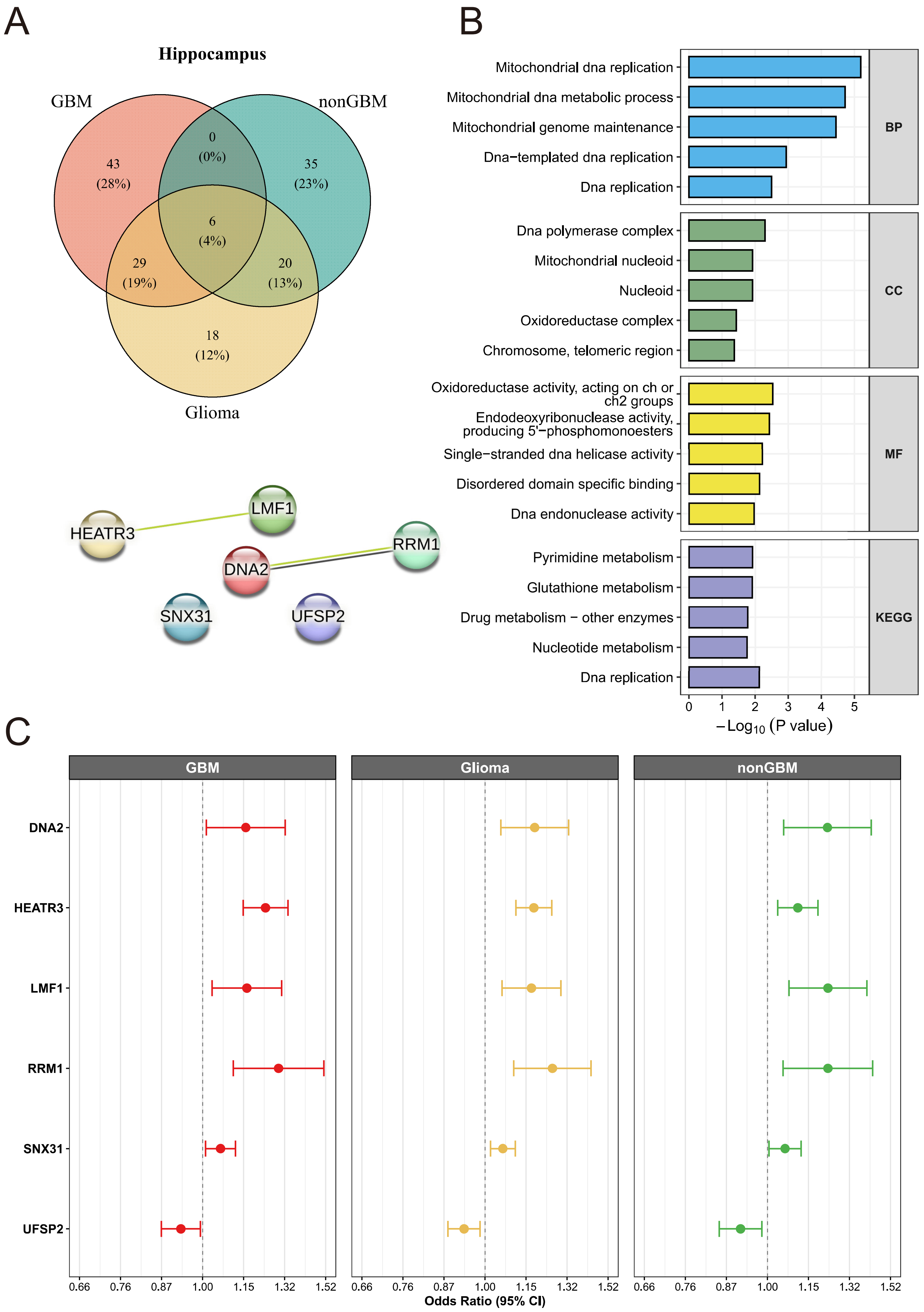

A
Hippocampus
GBM
nonGBM
Glioma
43 (28%)
0 (0%)
35 (23%)
6 (4%)
29 (19%)
20 (13%)
18 (12%)
HEATR3
LMF1
RRM1
DNA2
SNX31
UFSP2
B
Mitochondrial dna replication
Mitochondrial dna metabolic process
Mitochondrial genome maintenance
Dna−templated dna replication
Dna replication
BP
Dna polymerase complex
Mitochondrial nucleoid
Nucleoid
Oxidoreductase complex
Chromosome, telomeric region
CC
Oxidoreductase activity, acting on ch or ch2 groups
Endodeoxyribonuclease activity, producing 5'−phosphomonoesters
Single−stranded dna helicase activity
Disordered domain specific binding
Dna endonuclease activity
MF
Pyrimidine metabolism
Glutathione metabolism
Drug metabolism − other enzymes
Nucleotide metabolism
Dna replication
KEGG
0 1 2 3 4 5
−Log10 (P value)
C
GBM
Glioma
nonGBM
DNA2
HEATR3
LMF1
RRM1
SNX31
UFSP2
0.66 0.76 0.87 1.00 1.15 1.32 1.52
Odds Ratio (95% CI)

Figure 8

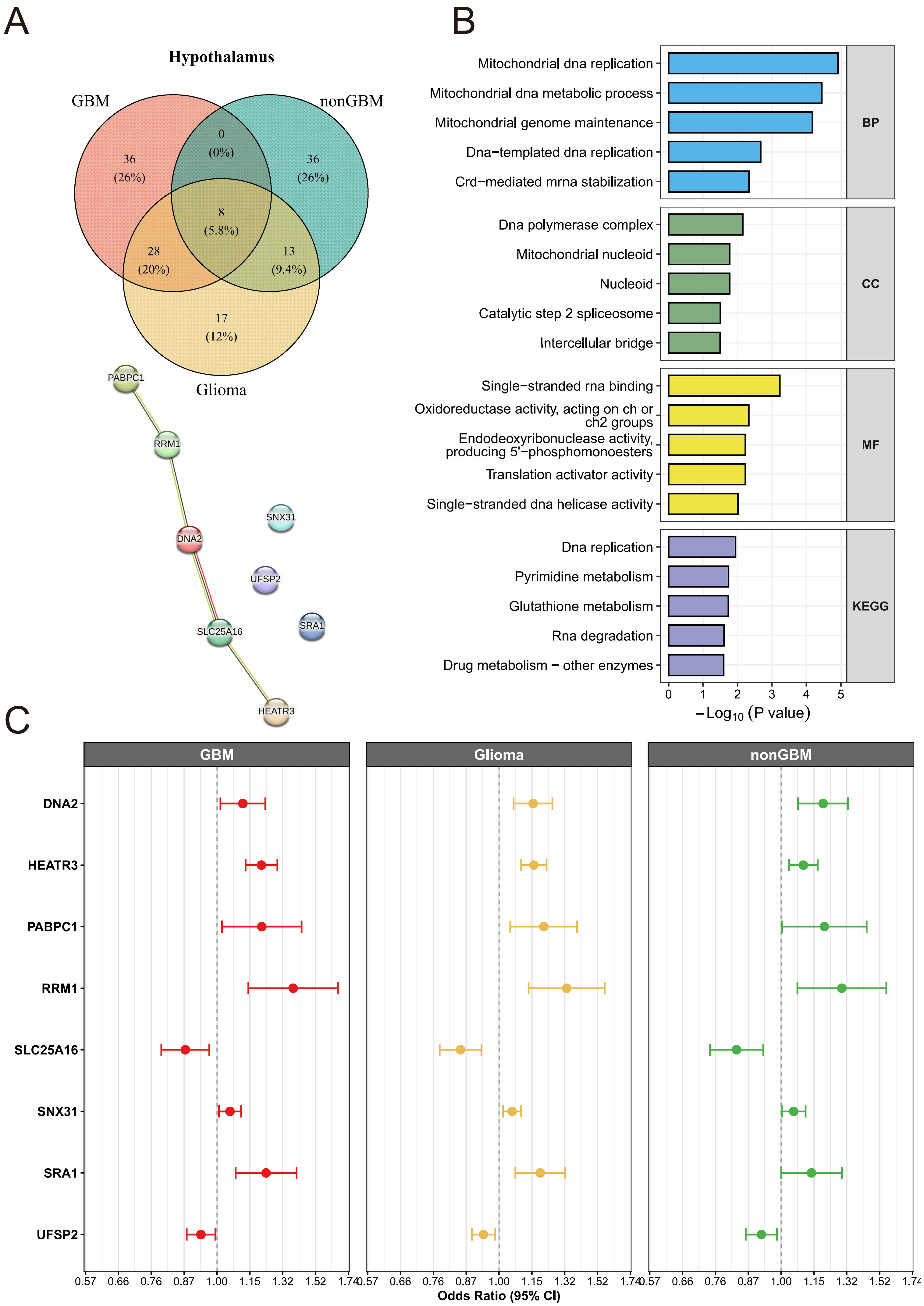
A
Hypothalamus
GBM
nonGBM
Glioma
36
(26%)
0
(0%)
36
(26%)
8
(5.8%)
28
(20%)
13
(9.4%)
17
(12%)
PABPC1
RRM1
DNA2
SLC25A16
HEATR3
SNX31
UFSP2
SRA1
B
Mitochondrial dna replication
Mitochondrial dna metabolic process
Mitochondrial genome maintenance
Dna−templated dna replication
Crd−mediated mrna stabilization
BP
Dna polymerase complex
Mitochondrial nucleoid
Nucleoid
Catalytic step 2 spliceosome
Intercellular bridge
CC
Single−stranded rna binding
Oxidoreductase activity, acting on ch or ch2 groups
Endodeoxyribonuclease activity, producing 5'−phosphomonoesters
Translation activator activity
Single−stranded dna helicase activity
MF
Dna replication
Pyrimidine metabolism
Glutathione metabolism
Rna degradation
Drug metabolism − other enzymes
KEGG
0 1 2 3 4 5
−Log10 (P value)
C
GBM
Glioma
nonGBM
DNA2
HEATR3
PABPC1
RRM1
SLC25A16
SNX31
SRA1
UFSP2
0.57 0.66 0.76 0.87 1.00 1.15 1.32 1.52 1.74
Odds Ratio (95% CI)

Figure 9

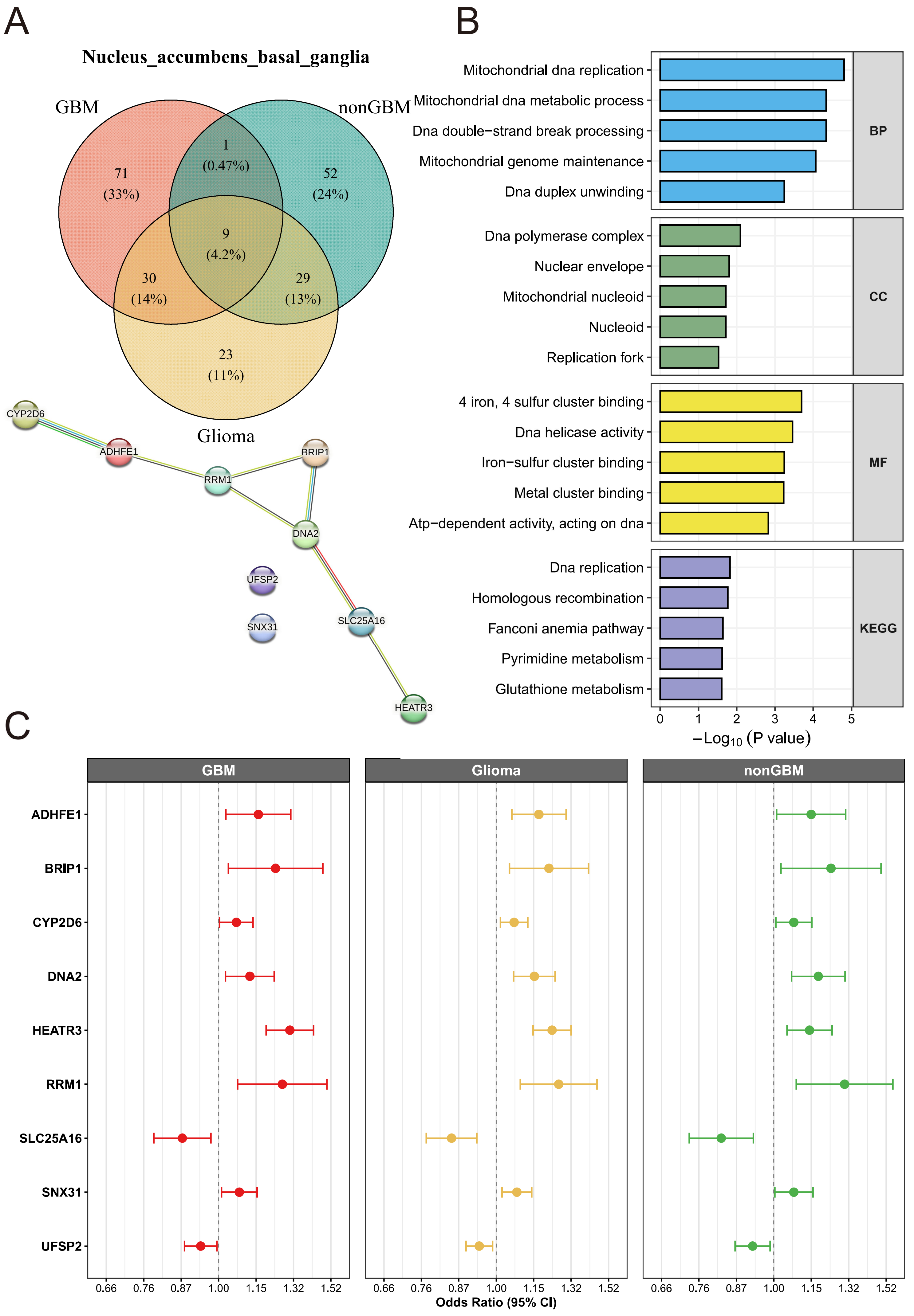
A
Nucleus_accumbens_basal_ganglia
GBM
nonGBM
Glioma
71 (33%)
1 (0.47%)
52 (24%)
9 (4.2%)
30 (14%)
29 (13%)
23 (11%)
CYP2D6
ADHFE1
RRM1
BRIP1
DNA2
UFSP2
SNX31
SLC25A16
HEATR3
B
Mitochondrial dna replication
Mitochondrial dna metabolic process
Dna double−strand break processing
Mitochondrial genome maintenance
Dna duplex unwinding
BP
Dna polymerase complex
Nuclear envelope
Mitochondrial nucleoid
Nucleoid
Replication fork
CC
4 iron, 4 sulfur cluster binding
Dna helicase activity
Iron−sulfur cluster binding
Metal cluster binding
Atp−dependent activity, acting on dna
MF
Dna replication
Homologous recombination
Fanconi anemia pathway
Pyrimidine metabolism
Glutathione metabolism
KEGG
0 1 2 3 4 5
−Log10 (P value)
C
GBM
Glioma
nonGBM
ADHFE1
BRIP1
CYP2D6
DNA2
HEATR3
RRM1
SLC25A16
SNX31
UFSP2
0.66 0.76 0.87 1.00 1.15 1.32 1.52
Odds Ratio (95% CI)

Figure 10

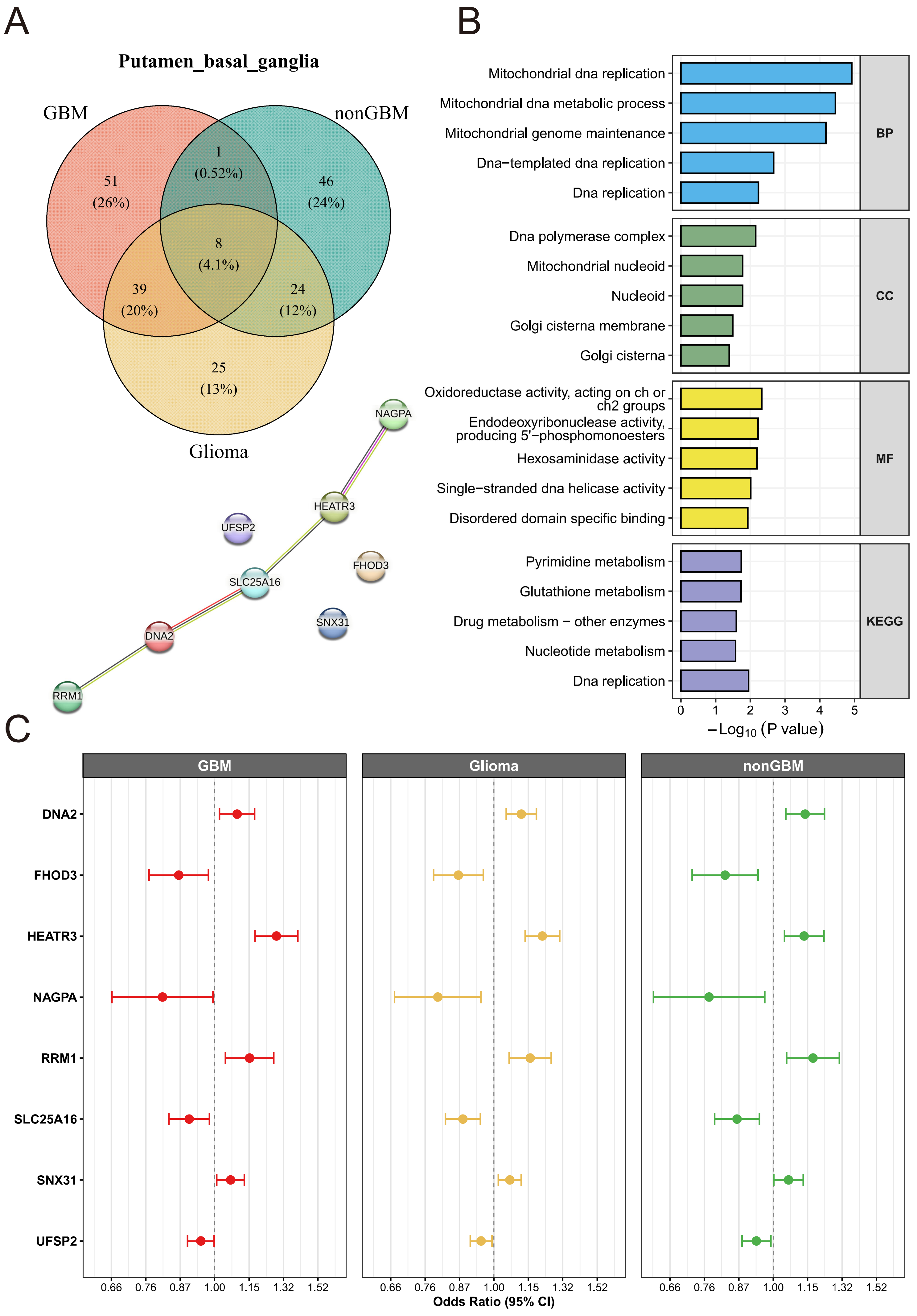
A
Putamen_basal_ganglia
GBM
nonGBM
Glioma
51
(26%)
1
(0.52%)
46
(24%)
8
(4.1%)
39
(20%)
24
(12%)
25
(13%)
NAGPA
HEATR3
UFSP2
FHOD3
SLC25A16
SNX31
DNA2
RRM1
B
Mitochondrial dna replication
Mitochondrial dna metabolic process
Mitochondrial genome maintenance
Dna−templated dna replication
Dna replication
BP
Dna polymerase complex
Mitochondrial nucleoid
Nucleoid
Golgi cisterna membrane
Golgi cisterna
CC
Oxidoreductase activity, acting on ch or ch2 groups
Endodeoxyribonuclease activity, producing 5'−phosphomonoesters
Hexosaminidase activity
Single−stranded dna helicase activity
Disordered domain specific binding
MF
Pyrimidine metabolism
Glutathione metabolism
Drug metabolism − other enzymes
Nucleotide metabolism
Dna replication
KEGG
0 1 2 3 4 5
−Log10 (P value)
C
GBM
Glioma
nonGBM
DNA2
FHOD3
HEATR3
NAGPA
RRM1
SLC25A16
SNX31
UFSP2
0.66 0.76 0.87 1.00 1.15 1.32 1.52
Odds Ratio (95% CI)

Figure 11

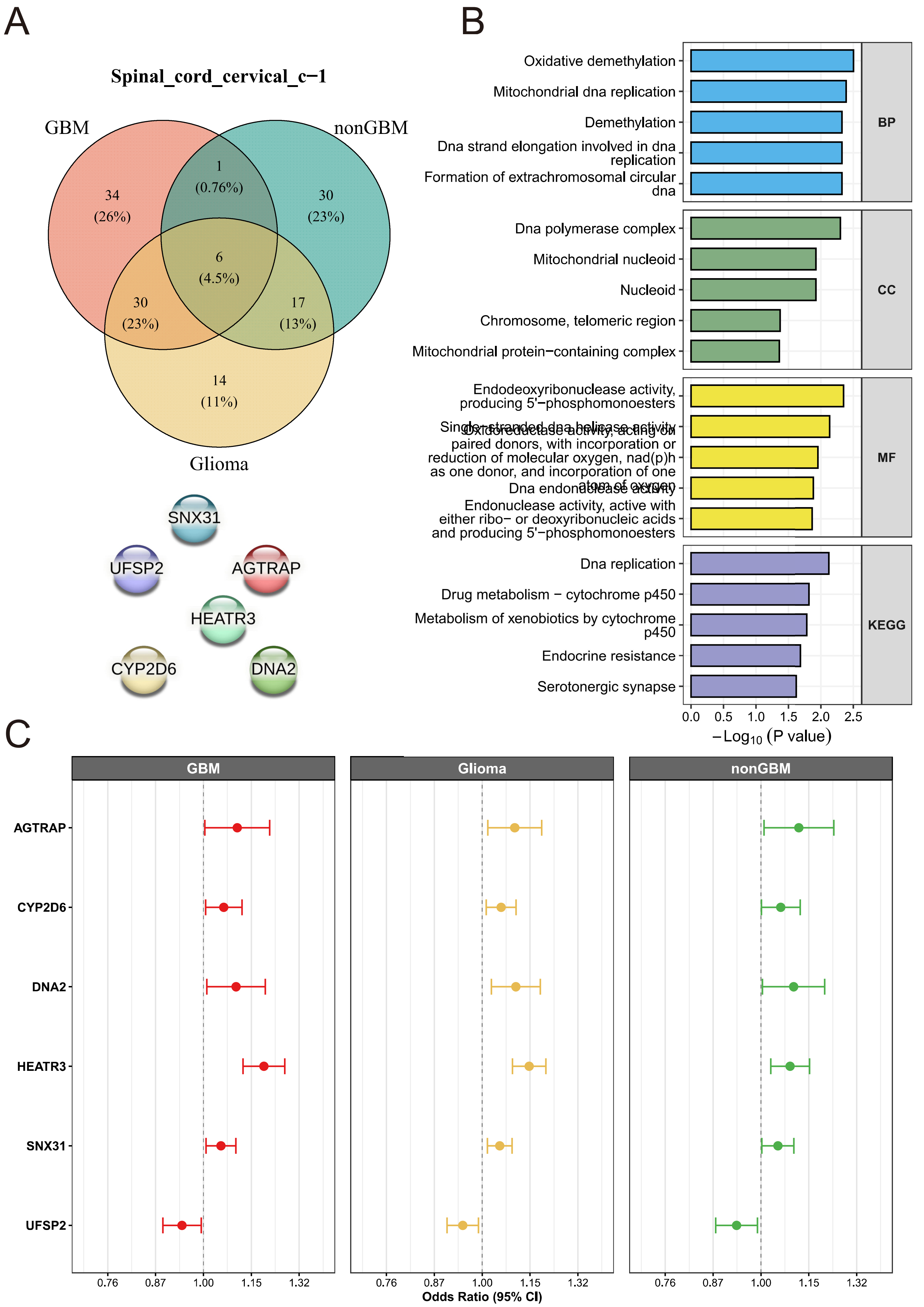
A
Spinal_cord_cervical_c−1
GBM
nonGBM
Glioma
34 (26%)
1 (0.76%)
30 (23%)
6 (4.5%)
30 (23%)
17 (13%)
14 (11%)
SNX31
UFSP2
AGTRAP
HEATR3
CYP2D6
DNA2
B
Oxidative demethylation
Mitochondrial dna replication
Demethylation
Dna strand elongation involved in dna replication
Formation of extrachromosomal circular dna
BP
Dna polymerase complex
Mitochondrial nucleoid
Nucleoid
Chromosome, telomeric region
Mitochondrial protein−containing complex
CC
Endodeoxyribonuclease activity, producing 5'−phosphomonoesters
Single−stranded dna helicase activity
Oxidoreductase activity, acting on paired donors, with incorporation or reduction of molecular oxygen, nad(p)h as one donor, and incorporation of one atom of oxygen
Dna endonuclease activity
Endonuclease activity, active with either ribo− or deoxyribonucleic acids and producing 5'−phosphomonoesters
MF
Dna replication
Drug metabolism − cytochrome p450
Metabolism of xenobiotics by cytochrome p450
Endocrine resistance
Serotonergic synapse
KEGG
0.0 0.5 1.0 1.5 2.0 2.5
−Log10 (P value)
C
GBM
Glioma
nonGBM
AGTRAP
CYP2D6
DNA2
HEATR3
SNX31
UFSP2
0.76 0.87 1.00 1.15 1.32
Odds Ratio (95% CI)

Figure 12

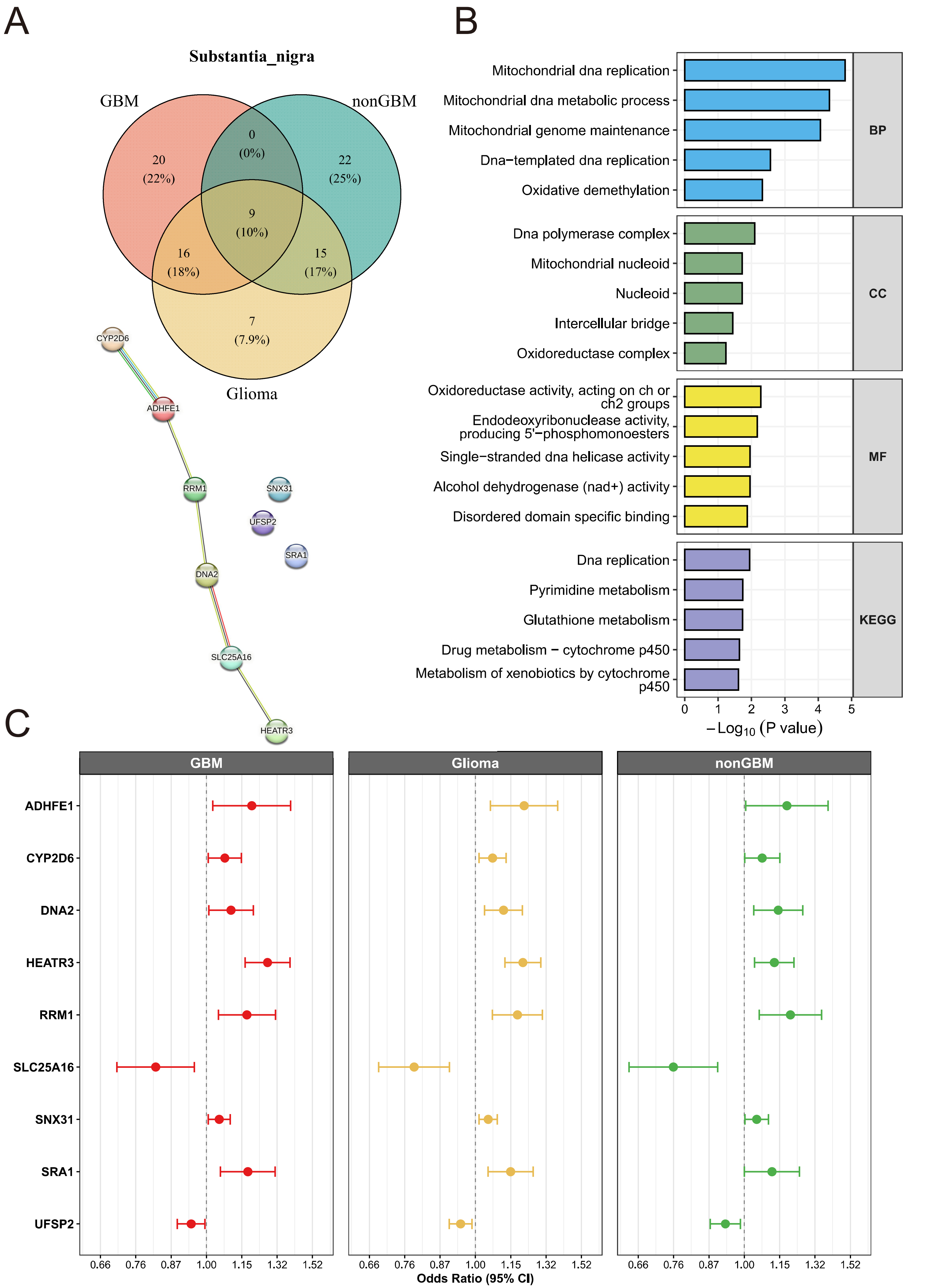
A
Substantia_nigra
GBM
nonGBM
Glioma
0
(0%)
20
(22%)
22
(25%)
9
(10%)
16
(18%)
15
(17%)
7
(7.9%)
CYP2D6
ADHFE1
RRM1
SNX31
UFSP2
SRA1
DNA2
SLC25A16
HEATR3
B
Mitochondrial dna replication
Mitochondrial dna metabolic process
Mitochondrial genome maintenance
Dna−templated dna replication
Oxidative demethylation
BP
Dna polymerase complex
Mitochondrial nucleoid
Nucleoid
Intercellular bridge
Oxidoreductase complex
CC
Oxidoreductase activity, acting on ch or ch2 groups
Endodeoxyribonuclease activity, producing 5'−phosphomonoesters
Single−stranded dna helicase activity
Alcohol dehydrogenase (nad+) activity
Disordered domain specific binding
MF
Dna replication
Pyrimidine metabolism
Glutathione metabolism
Drug metabolism − cytochrome p450
Metabolism of xenobiotics by cytochrome p450
KEGG
0 1 2 3 4 5
−Log10 (P value)
C
GBM
Glioma
nonGBM
ADHFE1
CYP2D6
DNA2
HEATR3
RRM1
SLC25A16
SNX31
SRA1
UFSP2
0.66 0.76 0.87 1.00 1.15 1.32 1.52
Odds Ratio (95% CI)
